# Neural circuit mechanisms for multi-tasking

**DOI:** 10.64898/2026.10.01.755960

**Published:** 2026-10-07

**Authors:** Manuel Beiran, Owen Marschall, Klavdia Zemlianova, Arianna Di Bernardo, Srdjan Ostojic, Ashok Litwin-Kumar

**Affiliations:** 1 Center for Theoretical Neuroscience, Zuckerman Mind Brain Behavior Institute, Columbia University, New York, NY USA; 2 Kavli Institute for Brain Science, Columbia University, New York, NY USA; 3 Laboratoire de Neurosciences Cognitives et Computationnelles, INSERM U960, École Normale Supérieure - PSL Research University, Paris, France

## Abstract

Neural activity during performance of a cognitive or motor task is often described as lying on a low-dimensional manifold in the space of all possible activity patterns. When multiple tasks are performed, an individual brain region’s activity may generate multiple such manifolds. Several models have been introduced to explain this capability, each with different assumptions concerning architecture and inputs. Here, we develop a theory of multi-tasking according to which models and experimental paradigms can be organized. We introduce a distinction between sequential and simultaneous multi-tasking, the performance of one task at a time or multiple in parallel. We show that interference between tasks often prevents multi-tasking and introduce a model of “task barcodes” that circumvents this problem and parameterizes a neuronal population’s task selectivity. Our theory reveals categorical distinctions between neural circuit mechanisms supporting multi-tasking that can be probed using physiological recordings.

## Introduction

The ability to generate different responses across changing goals and circumstances is a defining feature of intelligent behavior. This ability requires neural circuits to flexibly map sensory inputs to actions, across conditions with distinct behavioral demands. How such multi-tasking is achieved, without interference across conditions, remains an open question.

Simultaneous recordings of many neurons in animals trained to perform multiple tasks have characterized the geometry of neural activity associated with this behavioral flexibility. In primates^1–4^ and rodents^5,6^, different tasks, defined by specific combinations of stimuli and responses, are often associated with neural activity that lies in distinct regions of neural firing rate space. These regions are sometimes termed “task manifolds”^7,8^. Here, we ask how a single network can multi-task, that is, support multiple task manifolds each with different associated dynamics.

Numerous modeling studies have addressed aspects of this question, using different assumptions on the neural architecture that supports multi-tasking. Recurrent neural networks trained on multiple tasks frequently rely on explicit contextual inputs that modify a network’s dynamics depending on task demands^9–11^. Other studies have emphasized the role of preparatory activity that instructs a network to produce a specific behavior by setting an appropriate initial condition^12,13^. Theoretical studies have examined how changes in effective connectivity, mediated by mechanisms such as gating or synaptic modulation, can change a network’s dynamics^14–16^. Related ideas have also been explored in models of continual learning^17^ and contextual inference^18^. What are the relative advantages and limitations of each of these proposed mechanisms, and is each suitable for only certain behaviors or for any behavior that requires multi-tasking?

Here, we develop a theory that characterizes strategies a single recurrent network may employ to achieve multi-tasking. We propose a formal definition of what it means for a single network to multi-task without interference, and show that such interference frequently occurs for nonlinear recurrent neural networks. Our theory introduces a distinction between sequential multi-tasking, in which one task is performed at a time, and simultaneous multi-tasking, in which multiple are performed in parallel. We demonstrate that previous models of multi-tasking can be organized based on their mechanism for switching between tasks and avoiding interference. We also introduce a new model of “task barcodes” that limits interference and enables both simultaneous and sequential multi-tasking. Our analysis of this model identifies a novel relationship between the distribution of single-neuron task selectivities, which can be measured with neural population recordings, and a network’s ability to multi-task. We analyze concrete implementations of multi-tasking mechanisms and show that each produces qualitatively different features in neural firing rate dynamics. Finally, we describe how these mechanisms may be distinguished using neural data.

## Results

### Task variables and types of multi-tasking

Numerous studies have formalized the neural computations that support executing a single sensorimotor task, or solving a cognitive task, as dynamics operating on a set of *task variables*^9,10,19–21^ (Fig. 1A). These variables refer to quantities that are represented by neural activity, may evolve over time, and are required for task performance. While some variables may correspond to features of sensory inputs, others may be internally generated, such as neural representations of accumulated evidence, elapsed time, or goals. The ensemble of activity patterns that a neural system generates during one task is often described as lying on a low-dimensional *task manifold* in the space of neural firing rates, with a location on the manifold parameterized by the task variables (Fig. 1A, right). The dynamics of neural activity while the task is performed are represented by the flow field on this manifold. This geometrical language for describing the neural basis of task-related computation has become commonplace across theoretical and systems neuroscience^7,19,22,23^.

Individual neural circuits may support multiple sensorimotor tasks, for instance cycling and juggling (Fig. 1A). Each of these requires distinct processing of inputs, such as proprioceptive, vestibular, visual, or somatosensory signals, to generate motor commands. How does the picture of low-dimensional task manifolds extend to networks that multi-task? To answer this, we first distinguish between two types of multi-tasking. One is *sequential* multi-tasking, in which only one task is performed at a time, for example cycling at one moment and juggling at another (Fig. 1B, left). This notion is commonly applied when studying recurrent neural networks trained to perform one of a set of cognitive tasks at a time, given a task-specific contextual cue^9,10^. The second is *simultaneous* multi-tasking, which requires generating outputs for multiple tasks at the same time, for instance cycling and juggling at once (Fig. 1C, left). While this case is under-explored in theoretical studies, complex tasks often require simultaneously solving component subtasks, and some experimental paradigms explicitly demand simultaneous multi-tasking^24^.

In neural activity space, sequential multi-tasking corresponds to trajectories that switch from one manifold to another (Fig. 1B, right). Thus, the sequential multi-tasking manifold consists of a union of single-task manifolds with their associated flow fields. In contrast, for simultaneous multi-tasking, neural activity is not restricted to any single-task manifold. Instead, it represents all variables across the tasks currently being performed. For example, if tasks A and B are each defined by two variables, activity during the simultaneous performance of A and B lies on a four-dimensional manifold (Fig. 1C, right). Thus, the topology of the simultaneous multi-tasking manifold is that of the Cartesian product of the single-task manifolds. As we will show, different neural architectures may generate manifolds that support these two forms of multi-tasking.

### Implementing tasks through recurrent connectivity

Networks of interconnected neurons may implement task-related computations by generating dynamics through their recurrent interactions^19^. Recurrent neural networks are a popular model class for testing this hypothesis^25,26^, as they can, in principle, approximate arbitrary dynamical systems^27^. In these models, N neurons indexed by i=1…N are described by voltage-like variables $x_{i}$ and communicate through firing rates $\phi \left(x_{i}\right)$, where ϕ(x) is the activation function that maps x to firing rate (for our simulations, taken to be a rectified-linear function). The dynamics of individual neurons obey:

(1) $$\tau \frac{dx_{i}}{dt}=-x_{i}+\sum_{j=1}^{N}J_{ij}\phi \left(x_{j}\right)+I_{i}(t),$$

where $J_{ij}$ is the strength of the connection from neuron j to neuron $i,I_{i}$ is the external input received by neuron i, and τ is the membrane time constant.

For a network that performs a single task, a particularly direct relation between recurrent connectivity and low-dimensional task dynamics is obtained if the connectivity is assumed to take the form of a low-rank matrix^20,21,28^:

(2) $$J_{ij}=\frac{1}{N}\sum_{r=1}^{R}m_{i}^{r}n_{j}^{r}.$$

In this case, the dynamics defined by Eq. (1) lie in a subspace whose dimension is at most $R+R_{\text{input}}$ (where R is the rank of the matrix and $R_{\text{input}}$ is the dimension of the external input), which we call the *task subspace* (Fig. 2A, top). Activity is always attracted to this subspace if a network performs only one task. Transforming this activity through the firing rate nonlinearity produces a nonlinear task manifold whose intrinsic dimension equals that of the task subspace (Fig. 2A, bottom). Neural activity within the task subspace, or manifold, represents the variables $\vec{\kappa }$ and is the substrate for task-related computations (Fig. 1A). Linear readouts of this activity can produce time-varying outputs that represent solutions to cognitive or motor tasks^20^. Low-rank network models therefore provide an analytically tractable account of neural computation on low-dimensional subspaces (when measuring voltage) or manifolds (when measuring firing rate).

### Multi-tasking in recurrent neural networks and the problem of interference

To construct a multi-tasking network, we assume that J is a weighted sum of connectivity components related to different tasks and a non-task-related term that represents global inhibition (see Methods):

(3) $$J_{ij}=-g_{I}/N+\sum_{\mu =1}^{P}a_{\mu }J_{ij}^{\mu },\;J_{ij}^{\mu }=\frac{1}{N}\sum_{r=1}^{R_{\mu }}m_{i}^{r,\mu }n_{j}^{r,\mu }.$$

Here, P is the number of tasks, $a_{\mu }$ is the strength of the connectivity component associated with task μ, and $g_{I}$ is the magnitude of global inhibition. Each component $J^{\mu }$ is a rank-$R_{\mu }$ synaptic weight matrix, described by the connectivity vectors $m^{r,\mu }$ and $n^{r,\mu }$, representing a task associated with a manifold of dimension $R_{\mu }+R_{\text{input}}$. The assumption of summed connectivity components underlies the construction of many models of multi-task networks^13,15,29,30^. It can be traced to Hopfield networks, whose weight matrix is a sum of rank-one terms, each corresponding to an individual item to be memorized^31^. A similar structure arises naturally in models of sequential learning, where $J^{\mu }$ is the change in weights produced by learning of the μth task^32,33^.

The ability of a network to multi-task depends on how variables associated with different tasks interact. We begin by considering two immediate examples of recurrent neural networks that lack such interactions completely. The first is a network containing disjoint subsets of neurons, with each subset dedicated to a single task and lacking connections to any other. Such a model is at odds with the common observation of individual neurons exhibiting mixed selectivity to multiple tasks^7,34^. We therefore do not focus on this extreme further (although we will consider a relaxed form of task-specific selectivity later). The second example is a linear network in which each task is associated with an orthogonal subspace of firing rates, generated by orthogonal sets of connectivity vectors across tasks. In this situation, the dynamics within each subspace decouple, factorizing into single-task dynamics much like in networks with disjoint populations (see Appendix). However, the range of possible dynamics in linear networks is limited and does not include, for instance, multistability or asymptotically stable oscillations.

For nonlinear networks, interactions between task variables generically occur, even if the subspaces associated with different tasks are orthogonal (see Appendix). How do these interactions affect the multi-tasking ability of nonlinear networks whose connectivity is defined by Eq. (3)? To examine this, we constructed two-task networks whose connectivity vectors define orthogonal subspaces. Each connectivity component alone produces a limit cycle of a different frequency in the respective subspace (Fig. 2B). For a network whose synaptic weight matrix is the sum of these two components, it may seem that the dynamics in each subspace should decouple, as they do for linear networks. However, such a network produces only one oscillation in the task B subspace, while activity in the task A subspace is suppressed and occurs at an incorrect frequency (Fig. 2C). We use the term *task interference* to refer to interactions between task variables that prevent multi-tasking and say that task B interferes with task A in this example. Providing an external input aligned with either of the task subspaces does not prevent this limitation (Supp. Fig. S1).

For a network whose weight matrix is a sum of task-related components (Eq. 3), interference occurs if the connectivity vectors for each task are non-orthogonal, as expected. More surprisingly however, our mathematical analysis shows that even for orthogonal connectivity vectors, interactions between task variables often cause one or more task subspaces to become unstable. Thus, even if the dynamics constrained to the task A subspace are unchanged, small fluctuations in task B’s variables are amplified and subsequently suppress activity in the task A subspace, as in the example above. We call this type of interference a *transverse instability* of the task A subspace, as it is associated with unstable growth in the orthogonal direction corresponding to the task B subspace (Fig. 2D; compare with 2A, top). In the Appendix, we provide formal criteria for the existence and transverse stability of multi-tasking manifolds, and an analysis of these properties in networks of the form of Eq. (3). Whether a network satisfies these criteria depends on the structure of the weights and the activation function, and the analysis shows that straightforward extensions of nonlinear, low-rank recurrent neural networks to the multi-task setting (e.g. Fig. 2C, and more broadly any models in which the connectivity vectors are jointly Gaussian) generically lead to transverse instability. In subsequent sections, we describe neural circuit mechanisms that circumvent this general problem of task interference.

### Mechanisms for multi-tasking

#### Transient task-related activity

In the network of Fig. 2C, transverse instability implies that steady state activity occupies only one task manifold. One solution to this problem is to concern ourselves not with steady state activity, but instead only with activity that begins at some initial condition and subsequently evolves for a limited time. Selecting a task, then, requires setting an initial condition that transiently produces activity near the desired manifold^12^.

To more easily visualize the geometry of neural activity associated with multi-tasking, we studied a two-task setting in which task A requires the generation of a limit cycle as before and task B requires bistable dynamics (modeling, for instance, delay period activity following a cue taking one of two values that must be remembered; Fig. 3A). In this case, neural activity can be projected into a three-dimensional space, where the first two axes correspond to the task A subspace, and the third axis corresponds to the task B subspace. However, all of our results generalize to other combinations of tasks, and to different numbers of tasks (see Appendix).

Neural trajectories whose initial conditions overlap with the task B subspace approach one of the two fixed points, as desired (Fig. 3B). However, a limit cycle is produced only if the initial condition is perfectly aligned with the task A subspace. Any small deviation in initial condition along the task B subspace leads to movement toward one of the fixed points and a decaying oscillation. This is due to interference that produces transverse instability of the task A subspace (Fig. 3C; similar to Fig. 2D). We estimated how long task A is performed by measuring the time interval over which the projection of activity on the task A subspace exceeds some fraction of its initial value (Fig. 3C, blue dot). This interval grows as the initial condition is fine-tuned to minimize overlap with the task B subspace (Fig. 3D). However, the growth is at best logarithmic. Thus, extremely precise control of the initial condition is required to perform multiple tasks over long intervals.

Previous models of the production of multiple sequences of neural activity can be viewed as examples of transient multi-tasking, with the length of the recalled sequence proportional to the time interval over which the task is performed. Gillett et al.^13^ analyzed a model in which the rth element in sequence μ is associated with an activity pattern $m^{\mu ,r}$ and the connectivity component for each sequence is:

(4) $$J_{ij}^{\mu }\propto \sum_{r=1}^{R-1}m_{i}^{\mu ,r+1}m_{j}^{\mu ,r}.$$

This equation is a special case of Eq. 3, where each sequence μ is interpreted as a task and associated with activity in an R-dimensional manifold. An initial condition that overlaps with the first element of a sequence triggers the recall of the subsequent elements. However, as above, small overlaps with elements of other sequences produce interference, corresponding to the transverse instability of individual task subspaces^13,35^ (Supp. Fig. S2).

We conclude that transient solutions to sequential multi-tasking are viable for short time intervals, but longer time intervals require increasingly precise control of the initial condition by a preparatory process. Moreover, simultaneous multi-tasking is not easily achieved because an initial condition that has high overlap with multiple task subspaces leads to the task with the greatest degree of amplification quickly suppressing the others. We next investigate mechanisms that reduce this sensitivity to initial state.

#### Multistable task manifolds

Certain network models do not exhibit transverse instability and instead feature multiple stable task manifolds. Such multistable dynamics can be used as the substrate for sequential multi-tasking, with an initial condition set by a preparatory signal shifting the network state from the basin of attraction of one task manifold to another. Classical attractor models of place representations in multiple environments are an example of this^36–38^. In a single environment, place cells provide a spatial map commonly modeled as a continuous attractor. For a one-dimensional environment, the neural representation of space forms a manifold parameterized by one task variable. As an example, we assume a periodic encoding that forms a ring attractor^39^, analogous to the periodic encoding of two-dimensional environments by grid cells^40^. Place cells can remap across environments, and one hypothesis for this phenomenon is that a map of each environment is represented by an independent continuous attractor (Fig. 4A). A network that generates multiple maps can be obtained by summing weights corresponding to each map^36–38,41^.

This can be viewed as an example of multi-tasking in which each task requires a persistent representation of a location on a different map. In its simplest version, a ring attractor network with cosine-tuned synaptic weights is a rank-two recurrent neural network^42^, and adding weights of two such networks produces a rank-four network^38^. An analysis using the theory of low-rank networks shows that, for a range of parameters, this rank-four network exhibits bistability between two ring attractors embedded in mutually orthogonal subspaces^29^. Depending on the initial condition, activity converges to one of the two manifolds, an example of sequential multi-tasking (Fig. 4B).

This mechanism to generate multistability can also be applied to dynamic tasks (Fig. 4C). For this example, we studied the limit cycle and memory tasks of Fig. 3. Unlike the previous section, multistability requires a different construction of connectivity vectors: instead of Gaussian statistics, the elements of each vector follow the statistics of a cosine bump, as in ring attractor models^29^ (see Methods). This produces a network for which each task subspace has a basin of attraction in its vicinity (Fig. 4C). Preparatory inputs can then select tasks by moving the network state from one basin of attraction to another (Fig. 4D).

The cosine-tuned connectivity described above is a specific example of a synaptic weight distribution that prevents task interference, but how to avoid interference outside of this case is not yet clear. We developed a theory of the effect of synaptic weight distribution on the ability to sequentially or simultaneously multi-task that predicts the effects of cosine-tuned connectivity components (see Appendix). The theory also led us to introduce a simple mechanism that can be applied to arbitrary weight distributions to mitigate interference, which we will describe in a later section.

#### Modulation of effective connectivity

Other previous models of sequential multi-tasking have proposed modulation of the synaptic weight matrix as a mechanism for task selection^15,18,30,43^. In these models, it is assumed that task selection involves adjusting the coefficients $a_{\mu }$ in Eq. (3) to increase or decrease the strengths of connectivity components associated with different tasks (Fig. 5A). Unlike the previous sections, this mechanism implies that the flow field describing the evolution of neural firing rates changes upon the selection of a new task, so that selecting a task stabilizes the corresponding manifold.

An extreme version of this mechanism that sets $a_{\mu }=1$ for a selected task and $a_{\nu \neq \mu }=0$ for all other tasks enables sequential multi-tasking by completely overwriting the synaptic weight matrix each time a new task is selected. Several models make this assumption^15,18^. However, recent work has shown that such dramatic alterations in weights are not required in nonlinear networks^30^. Instead, to select task μ, modestly increasing only $a_{\mu }$ suffices. The amount of increase required depends on the distribution of $a_{\nu \neq \mu }$ as well as the number of tasks P relative to the network size N. Thus, nonlinear cross-task interference aids rather than hinders multi-tasking by allowing the selected task’s dynamics to suppress those of unselected tasks.

When a task is selected through this mechanism, the associated manifold is globally attracting. This permits multi-tasking through sequential modulation of the $a_{\mu }$ associated with different tasks. Fig. 5B illustrates selection of task A (left) followed by task B (right) by increases in $a_{A}$ and $a_{B}$. However, such a mechanism alone does not enable simultaneous multi-tasking.

What biophysical processes may support task-related changes in effective connectivity? Previous studies have suggested that neuromodulation^43^ or short-term synaptic plasticity may modify the effective weight between pairs of neurons, although ensuring that these modifications are specific to individual connectivity components may be challenging^30^. An attractive interpretation associates each connectivity component with distinct corticothalamic loops whose gains are selectively modulated, for instance by the activity of basal ganglia output nuclei, to select a task^15^ (Fig. 5C).

#### Selection by a rule input

Transient activity or modulation of effective connectivity underlie many mathematical models of multi-task activity^12,15,30,35^. However, recurrent neural networks trained to perform flexible computations through gradient descent typically rely on a different mechanism. A tonic external input, sometimes called a “rule” input, excites or inhibits a task-specific pattern of neurons and switches each time a new task is selected^9,10^ (Fig. 6A). Like in the previous section, this implies that the flow field changes upon task selection, but this change is not due to direct modulation of the weight matrix but rather the nonlinear effect of the rule input.

A recurrent neural network trained in this manner, without a constraint on the rank of its weight matrix, successfully performs the limit cycle and memory tasks examined in Figs. 3–5. Neural activity projected onto subspaces defined by the top principal components of activity during performance of each task follows trajectories similar to those of previous mechanisms (Fig. 6B). These task subspaces are nearly orthogonal (mean subspace angle of 80 ± 6 degrees across 10 trained networks) and account for a large fraction of the variance of neural activity during performance of each task (0.85 ± 0.1 for task A and 0.994 ± 0.001 for task B). These properties of orthogonality and low dimensionality of task subspaces in trained networks are similar to those of the low-rank networks we have examined so far, although the training procedure did not explicitly require low-rank weight updates or low-dimensional activity.

However, the distribution of individual neuron selectivities to the two tasks differed from previous mechanisms. We computed a task selectivity index ranging from −1 (purely modulated by task B) to 1 (purely modulated by task A). Neurons exhibited a range of selectivities, but a much larger fraction had values near the extremes as compared to, for example, networks that multi-task through modulation of effective connectivity (Fig. 6C). This observation is consistent with prior studies that report the emergence of task-specific modules of neurons over gradient descent training^9,10^.

#### Barcoded connectivity as a solvable model of multi-tasking

Although rule inputs thus support multi-tasking in networks trained through gradient descent, an analytically tractable model of this task selection mechanism is not yet available (Supp. Fig. S1; see Appendix). We therefore developed a new multi-task network model, motivated by the high selectivity of neurons in trained networks (Fig. 6C) and the fact that recurrent neural networks with groups of neurons whose connectivity statistics differ are more flexible in producing time-varying activity^16,21^. In the model, a quantity $b_{i}^{\mu }$ describes the degree to which neuron i’s connections are tuned to task μ (Fig. 7A, top). We call the vector $b^{\mu }$ the task μ “barcode.” The connectivity vectors $m^{\mu ,r}$ and $n^{\mu ,r}$ in Eq. 3 are then defined as

(5) $$m_{i}^{\mu ,r}=b_{i}^{\mu }\cdot m_{\text{ind},i}^{\mu ,r},\;n_{i}^{\mu ,r}=b_{i}^{\mu }\cdot n_{\text{ind},i}^{\mu ,r}.$$

The elements of $m_{\text{ind}}^{\mu ,r}$ and $n_{\text{ind}}^{\mu ,r}$ are random variables drawn independently for each neuron, as in previous sections. Unlike before, these elements are then multiplied by the barcode to generate the connectivity component for task μ (Eq. 3). To begin, we will assume that the barcode $b^{\mu }$ is a binary vector in which a random fraction f of elements, chosen independently for each task, are nonzero (we generalize this in the Appendix). Each connectivity component $J^{\mu }$ is additionally scaled by 1/f, which ensures that single-task dynamics are preserved across f. In this model, the barcode can be interpreted as defining those neurons whose synapses are modified so that the network can perform task μ (Fig. 7A, bottom). Because a neuron’s selectivity to each task is drawn independently, this model does not imply the existence of disjoint clusters of similarly selective neurons.

The introduction of barcodes enables rule inputs that are aligned to each task’s barcode to switch a network’s activity between task manifolds (Fig. 7B,C). A mathematical analysis demonstrates that this ability is due to the rule input selectively modulating the gain of the neuronal subpopulation defined by the selected task’s barcode (see Appendix). It is also possible to provide a rule input that selects multiple tasks at once if it is aligned with multiple barcodes, enabling simultaneous multi-tasking (Fig. 7D; see Appendix). In this case, neural trajectories both oscillate in the task A subspace and maintain a separation along the task B subspace, although the magnitude of each projection is moderately reduced compared to the single-task case. Thus, a single network may either sequentially or simultaneously multi-task, a flexibility that, to our knowledge, has not been explored in previous models.

One concern is that, if f is small (relative to 1/P), the model approaches the unrealistic extreme of disjoint task-specific populations that we rejected earlier. However, our theoretical analysis shows that very low values are not required. In fact, a fixed value of f, that does not decrease as the number of tasks P grows, is sufficient (see Appendix). This means that, in networks that support many tasks, individual neurons each participate in many tasks. As an example, we constructed a network with f=0.3 that multi-tasks between 12 tasks that each require oscillatory, sequential, or fixed-point dynamics (Fig. 7E, top). Application of rule inputs, aligned either with a single barcode or multiple, enables sequential multi-tasking of individual tasks or simultaneous multi-tasking, respectively. Neuronal firing rates are heterogeneous and cannot be clustered into discrete response profile categories (Fig. 7E, bottom). The model therefore demonstrates that increased task selectivity, and the additional flexibility it provides for generating dynamics, does not require disjoint task-specific subpopulations of neurons.

## Discussion

### Comparison of multi-tasking mechanisms

Table 1 summarizes the four multi-tasking mechanisms we have described. Although new mechanisms may be introduced, categorical distinctions between those we have described are revealed. The first concerns the capacity for simultaneous multi-tasking, which is only enabled by rule inputs in the models we have considered (Table 1, second column). Another concerns the nature of the flow field that determines the neural dynamics (Table 1, third column). If tasks are associated with either transient or multistable activity, this flow field is unchanged across tasks, meaning that task selection must be achieved through a preparatory process that determines the initial state. Moreover, only in the case of multistability are multiple stable attractors corresponding to different tasks present at once. On the other hand, task selection through a change in effective connectivity or a rule input implies that the flow field is modified upon task selection. We note that these mechanisms are not mutually exclusive. For example, barcoded connectivity could enable modulation of effective connectivity to support simultaneous multi-tasking, or reduce the precision of initial condition required for transient task selection.

In many of the models we examined, the mechanism that supports multi-tasking is associated with certain parameters that must be adjusted to combat the problem of interference (Table 1, fourth column). For task selection associated with transient activity, this parameter is the precision of the initial state. For modifications of effective connectivity, it is the magnitude of change in the connectivity component associated with the selected task. For models with barcoded connectivity, it is the degree of task selectivity, as measured in our model by the barcode sparsity f. Our mathematical analysis shows that, as task diversity (as quantified by the degrees of amplification associated with each task) and thus the potential for interference increases, each of these parameters must be adjusted more to compensate (see Appendix).

### Model simplifications and extensions

We have made several simplifying assumptions to derive our theory. One is to assume that each task corresponds to an independent computation and is associated with an orthogonal subspace in neural firing rate space. This assumption is made by many theoretical models^13,15,18,30^. Experiments with multiple task epochs, such as fixation, delay, and response periods, may be best thought of as a series of tasks, each corresponding to a single such epoch, in our model^18^. However, certain tasks may instead be described as parametrically related to one or multiple others, for instance adjusting the speed or timing of a motor program^44^. In this case, one may expect the overlap between task subspaces to smoothly depend on this parameter^45^. Alternatively, such situations may be conceived as a single, higher-dimensional task in which the parameter is identified with a new task variable.

We also focused on neural networks that generate time-varying, nonlinear dynamics, which lead to the phenomenon of interference that our theory examines (Fig. 2D). Neurons themselves are nonlinear, and restricting to linear dynamics greatly limits the range of computations that can be performed, arguing against models of neural dynamics that are exclusively linear. However, it should be noted that for brain regions that primarily respond to external inputs from other regions, rather than generating sustained patterns of activity, amplification that leads to task interference may be weak. We therefore predict that the mechanisms we describe are particularly important for brain regions that support working memory and motor pattern generation.

For simplicity, most of our examples have illustrated multi-tasking with two tasks. Our theory generalizes to any finite number of tasks P, in the limit N≫P (see Appendix). When P is comparable to N, more analysis is required, and theoretical results exist only for certain cases. These include analyses of capacity for storage of fixed-point or continuous attractors^31,37,46^. For storage of attractors or sequences with asymmetric connectivity, the limit of large P may be associated with the emergence of chaotic activity due to interference between tasks^13,47^. A similar phenomenon was analyzed in a recent study that focused on multi-tasking through changes in effective connectivity^30^. Defining and calculating a general notion of capacity for multi-tasking recurrent neural networks is an open problem.

Our analysis focuses on dynamics on task manifolds while remaining agnostic as to the readout of these dynamics. Distinct tasks may be associated with distinct linear readouts, or the weights of a common readout may overlap with multiple task manifolds. Alternatively, readout-related activity may correspond to a dimension in neural activity space that is shared across tasks but does not interfere with each task’s recurrent dynamics (see Appendix).

As shown in the Appendix, only one task is transversely stable and all the rest are unstable if the elements of the connectivity vectors, $m_{i}^{\mu }$ and $n_{i}^{\mu }$, are drawn independently from a jointly Gaussian distribution, as in many low-rank network models^20,30,48^. Task barcodes (Fig. 7) are a parsimonious relaxation of this Gaussian assumption and consistent with observations of modular neural subpopulations in experimental recordings^49,50^ and task-trained networks^9,10^. In models of continual learning, barcodes may be produced by a task-specific signal that selects a random subset of neurons for synaptic weight changes and does not require pre-allocation of neuronal subpopulations for distinct tasks. However, other non-Gaussian distributions of connectivity vector elements, for example cosine-tuned connectivity vectors^29,38^ (Fig. 4), can also support multi-tasking. We provide a general mathematical analysis of the effects of these distributions on multi-tasking in the Appendix.

### Sequential and simultaneous multi-tasking in experiments

Forms of sequential multi-tasking have been examined in various experimental paradigms. These include the production of different movement patterns in trained visuomotor tasks in primates^51–53^ and rodents^54^. Natural behavior is often viewed as a sequence of distinct motor patterns^4,55–58^. Many of these studies report the existence of distinct task subspaces^4,51,53,58^ and high neuronal task selectivity^54,57^, consistent with our models.

Although naturalistic behavior often involves the generation of distinct movement patterns across different effectors, simultaneous multi-tasking has been studied relatively less frequently in experiments. The most common examples are forms of memory-guided tasks in which multiple items must be stored in memory at once. Recent studies have suggested that, in such situations, each item may be stored in a largely orthogonal subspace, or “slot”^59–62^. Executing multiple tasks at once often degrades performance, a phenomenon known as dual-task interference^63^. Recordings in primate lateral prefrontal cortex demonstrate that this interference is associated with neurons that participate in both tasks^24^, consistent with our analysis which shows that high single-neuron task selectivity supports simultaneous multi-tasking (Fig. 7).

A system capable of sequential or simultaneous multi-tasking can be viewed as a compositional system, in that it can perform computations in different orders or combinations^64^. Indeed, trained animal behaviors that involve sequential^51–53^ or simultaneous^65^ multi-tasking are sometimes described as compositional. We have focused on how a single neural network performs multiple distinct computations through a selection mechanism, which is a prerequisite for compositional behaviors unless each computation is associated with a dedicated subset of neurons. A complete understanding of such behaviors will also require an understanding of the systems that represent rules dictating which computations should be selected and when. Some forms of composition may also involve rules for combining computations that extend beyond sequential or simultaneous multi-tasking^64,66^.

### Signatures of multi-tasking mechanisms in neural data

In experiments with explicit task structure, task subspaces may be identified by performing dimensionality reduction on independent subsets of data. An unsupervised method for identifying putative task-dependent factors was recently proposed that may be applied to experiments without such explicit structure^67^. Several studies have quantified the overlap of identified task subspaces, reporting increased alignment for more similar tasks. This may reflect similarity of the stimulus features^53^, sensorimotor transformations^45,51^, or muscle groups^68^ required for task performance. The degree of subspace alignment varies across brain regions: sensory areas tend to reuse subspaces across tasks, while cognitive and motor regions tend to orthogonalize^4^. In higher regions, certain task dimensions have been identified as responsible for executing components of a given task that may be shared with other tasks or task conditions^53^. A recent study suggests that motor cortex may combine “subskills” whose associated motor readouts additively produce a desired trajectory, a form of simultaneous multi-tasking if each subskill is identified as a single task^51^. Activity across epochs each requiring multiple such subskills may therefore span non-orthogonal subspaces^45,51,68^.

Methods for inferring the flow field associated with neural dynamics have been proposed^69^, some of which involve fitting low-rank recurrent neural networks to data^70^. Evaluating whether the flow field is task-dependent, in regions of neural state space that are explored in multiple tasks, will distinguish mechanisms that rely on initial conditions or changes in the flow field for task selection. Optogenetic perturbations that disrupt preparatory activity and bias activity away from one task manifold and toward another may also be used to probe this distinction. O’Shea et al.^71^ studied the effects of optogenetic and electrical microstimulation perturbations to activity in primate motor cortex during a reaching task. This revealed activity consistent with a low-dimensional, transversely stable subspace. This provides evidence against the transient mechanism we described, though it does not immediately distinguish between other mechanisms.

The degree to which individual neurons are tuned to specific task variables or exhibit mixed selectivity to all variables has been debated^34,50,72,73^. Analyses of feedforward networks have demonstrated that mixed selectivity to multiple task variables increases the dimension of a neural representation and the linear discriminability of distinct stimulus classes^34^. Analysis of neuronal selectivity across many mouse brain areas found that such mixing is common and did not identify evidence for clustering into discrete functional neuron types^74^. In contrast, analyses of recurrent models show that subpopulations of neurons with structured selectivity to distinct variables increase the flexibility with which a network can generate dynamics^16,21^.

Our model of task barcodes suggests that when an individual network is responsible for generating very different dynamics, the required degree of task selectivity increases. It also suggests that neuronal populations selective to tasks that are more prone to interference may have reduced overlap. In this model, task variable encoding is distributed across neurons but the degree of selectivity is continuously parameterized by the barcode sparsity. Thus, the model permits interpolation between the extremes of disjoint task-specific subpopulations and fully distributed representations. Moreover, because a neuron’s selectivity to each task is drawn independently, the model does not predict clusters of neurons with identical selectivities. More broadly, our analysis identifies the distribution of task selectivities across a neural population as an important determinant of its multi-tasking capability.

## Methods

### Network dynamics

We consider a recurrent neural network of N firing rate neurons, whose dynamics and connectivity are defined by Eqs. (1) and (3). A low-dimensional description of the network dynamics can be obtained by projecting neural activity $x_{i}$ onto the connectivity vectors associated with each task and the mean activity direction:

(6) $$\kappa_{r,\mu }=\frac{1}{N}\sum_{i=1}^{N}m_{i}^{r,\mu }x_{i},\;\overset{‾}{x}=\frac{1}{N}\sum_{i=1}^{N}x_{i}.$$

In the Appendix, we analyze the dynamics of these projections in the limit of large N using the theory of low-rank recurrent neural networks.

### Task-related connectivity

We generate task-related connectivity components by specifying the distributions of the connectivity vector elements $m_{i}^{\mu }$ and $n_{i}^{\mu }$ in Eq. (2). For the bistable working-memory task, we use a rank-one component:

(7) $$J_{ij}^{\mu }=\frac{1}{N}m_{i}^{\mu }n_{j}^{\mu },$$

where $m_{i}^{\mu }$ and $n_{i}^{\mu }$ are sampled from a Gaussian distribution with zero mean and covariance $\sigma_{\mu }$ unless otherwise specified.

For the generation of limit cycle dynamics, we use a rank-two component

(8) $$J_{ij}^{\mu }=\frac{1}{N}\left(m_{i}^{1,\mu }n_{j}^{1,\mu }+m_{i}^{2,\mu }n_{j}^{2,\mu }\right),$$

where the elements of the four connectivity vectors $\left(m_{i}^{1,\mu },n_{i}^{1,\mu },m_{i}^{2,\mu },n_{i}^{2,\mu }\right)$ are drawn from a four-dimensional Gaussian distribution, unless otherwise specified, with zero mean and covariance ${\left(\sigma_{\mu }\right)}_{rr^{\prime }}=\text{Cov}\left(n_{i}^{r,\mu },m_{i}^{r^{\prime },\mu }\right)$ :

(9) $$\sigma_{\mu }=\left(\begin{array}{cc} \eta_{\mu } & \omega_{\mu }\\ -\omega_{\mu } & \eta_{\mu } \end{array}\right).$$

All other pairs of connectivity vector elements, including those of different tasks, are uncorrelated. For each task, the variance of $m_{i}^{\mu }$ is set to one and the variance of $n_{i}^{\mu }$ is set to a constant such that the covariance matrix defining the distribution of all connectivity vector elements is positive definite. Note that the value of this constant does not affect the dynamics in the large N limit.

In Fig. 2, we study the dynamics of a network with two connectivity components each associated with limit cycle dynamics. The task parameters are $\eta_{A}=1.3,\omega_{A}=0.45$ for task A and $\eta_{B}=1.7,\omega_{B}=1.7$ for task B. The network has N=1000 neurons with a rectified-linear activation function ϕ(x)=max(x-θ,0) with threshold θ=-1. The task outputs are projections of the firing rates ϕ(x) onto the readout vectors $m^{1,A}/(0.8N)$ and $m^{1,B}/(3N)$, respectively.

### Models of task selection

#### Transient activity.

The network of Fig. 3B,C contains N=10,000 neurons with rectified-linear activation functions. The threshold is θ=-1, and $g_{I}=2$. Task A is associated with limit cycle dynamics with $\eta_{A}=1.6,\omega_{A}=0.8$, and task B is associated with bistable dynamics with $\sigma_{B}=1.8$. Task selection is determined by the initial condition. For task B, the initial condition is $x(0)=\pm 0.25m^{B}+\overset{‾}{x}(0)1$, where $\overset{‾}{x}(0)$ is determined as the self-consistent solution to $\overset{‾}{x}(0)=-\frac{g_{I}}{N}\sum_{i=1}^{N}\phi \left(x_{i}(0)\right)$. For task A, it is $x(0)=\left(m^{1,A}+m^{2,A}\right)/2+\overset{‾}{x}(0)1$. Trajectories correspond to 60 time units.

The network of Fig. 3D is similar to the above except $\sigma_{B}=1.7$. The initial conditions are $x(0)=\left(m^{1,A}+m^{2,A}+cm^{B}\right)/2+\overset{‾}{x}1$, where c determines the task B projection. The simulation is repeated for c=±|c|, and the reported duration is the minimum across these two repetitions.

#### Multistability.

In Fig. 4A,B, we study a network with two connectivity components supporting ring-attractor dynamics, with N=10,000 neurons with rectified-linear activation functions and θ=-1. For each task, preferred angles $\psi_{i}^{\mu }$ are assigned to each neuron by randomly permuting equally spaced angles on [0,2π). The synaptic weight matrix is

$$J_{ij}=\frac{a_{A}}{N}\text{cos}\left(\psi_{i}^{A}-\psi_{j}^{A}\right)+\frac{a_{B}}{N}\text{cos}\left(\psi_{i}^{B}-\psi_{j}^{B}\right),$$

with $a_{A}=2.5$ and $a_{B}=2.3$. Thus, the connectivity patterns are cosine- and sine-distributed.

In Fig. 4C,D, a rank-two limit cycle component is combined with a rank-one component supporting fixed points in a network of N=1000 neurons with the same activation function. The synaptic weight matrix is

$$J_{ij}=\frac{a_{A}}{N}\text{cos}\left(\psi_{i}^{A}-\psi_{j}^{A}-\delta \right)+\frac{a_{B}}{N}\text{cos}\;\psi_{i}^{B}\;\text{cos}\;\psi_{j}^{B},$$

with $\delta =0.35\pi ,a_{A}=2.2/(\text{cos}\;\delta )$, and $a_{B}=2.6$. The two trajectories share an initial condition and receive opposite instantaneous perturbations $\Delta x=\pm 1.2m_{B}$ at t≃19.8, where $m_{B}=\sqrt{2}\;\text{cos}\;\psi^{B}$. Readouts are projections of ϕ(x) onto the corresponding task patterns, normalized by N. The task A pattern is defined as $m^{A}=\sqrt{2}\;\text{cos}\;\psi^{A}$, while the task B pattern was first defined as $m^{B}=\sqrt{2}\;\text{cos}\;\psi^{B}$ and was subsequently orthogonalized with respect to patterns $\text{cos}\;\psi^{A}$ and $\text{sin}\;\psi^{A}$. The perturbation was also provided along this orthogonalized B pattern.

#### Modulation of effective connectivity.

The parameters of the network in Fig. 5 are the same as those of Fig. 3. Task selection is achieved by setting $a_{\mu }=1$ for the selected task and $a_{\mu }=0.5$ for the unselected task. The initial condition $x_{i}(0)$ when task A is selected is drawn independently from a standard Gaussian distribution. When task B is selected, for the first 10 time units, an external input $I_{i}(t)=\pm m_{i}^{B}e^{-t/10}$ perturbs the trajectory toward one of the two fixed points. Trajectories correspond to 60 time units.

#### Task-trained network with rule inputs.

The network of Fig. 6 contains N=500 neurons with rectified-linear activation functions and thresholds $\theta_{i}$. Activity evolves according to:

(10) $$x(t+\Delta t)=(1-\Delta t)x(t)+\Delta t\left(J\phi (t)+W_{\text{in}}h(t)\right),\;y(t)=W_{\text{out}}\phi (t),$$

where the length-4 vector h(t) denotes external input channels, $W_{\text{in}}$ is the N×4 input weight matrix, $W_{\text{out}}$ is the 2×N output weight matrix, $y={\left(y_{A},y_{B}\right)}^{⊤}$ denotes linear readouts for tasks A and B, and Δt=0.1. The elements of each weight matrix are initialized as independent Gaussian variables with standard deviations $1.5/\sqrt{N}$ for J, 0.5 for $W_{\text{in}}$, and $0.1/\sqrt{N}$ for $W_{\text{out}}$. Thresholds are initialized as $\theta_{i}=-0.2$ for each neuron.

The four input channels $h=\left(h_{A},h_{B},h_{1},h_{2}\right)$ consist of two binary rule channels $h_{A}$ and $h_{B}$ that are nonzero when task A or B is selected and two binary stimulus channels $h_{1}$ and $h_{2}$, one of which is nonzero during the interval t∈[1, 2) for task B trials. Inputs depicted in Fig. 6A are determined by the columns of $W_{\text{in}}h$. Each trial is of length 60 time units, and x(0)=0. For task A trials, the target output is $y_{A}^{*}(t)=\text{sin}(2\pi \cdot 0.25t)$. For task B trials, the target output is $y_{B}^{*}=+1$ if the network receives input $h_{1}$ or $y_{B}^{*}=-1$ if the network receives input $h_{2}$.

Networks are trained using backpropagation through time and the Adam optimizer to minimize the mean squared difference between $y_{\mu }(t)$ and $y_{\mu }^{*}(t)$ computed over the interval t>5, where μ denotes the current trial’s task. All parameters $J,W_{\text{in}},\theta$, and $W_{\text{out}}$ are optimized. Each training batch contains 3 trials, one each of task A, task B with input 1, and task B with input 2. During training, the learning rate exponentially decays from $10^{-3}$ to $10^{-4}$ over 1000 epochs, and gradients are clipped to a maximum norm of 1.

#### Barcodes.

The network of Fig. 7B–D is similar to those of Figs. 3 and 5, except that the connectivity is constructed using the barcode model (Eq. 5) with $f=0.7,\eta_{A}=1.75,\omega_{A}=1$, and $\sigma_{B}=1.7$. The input to the network is $I(t)=I^{\text{rule}}(t)+I^{\text{stim}}(t)$, where $I_{i}^{\text{rule}}(t)=sb_{i}^{\mu }$ to select task μ and $I_{i}^{\text{rule}}(t)=s\cdot \text{max}\left(b_{i}^{A},b_{i}^{B}\right)$ to select both tasks simultaneously, and s=2. As in Fig. 5, the initial condition $x_{i}(0)$ when task A is selected is drawn independently across neurons from a standard Gaussian distribution, and when task B is selected, for the first 10 time units, a stimulus input $I_{i}^{\text{stim}}(t)=\pm m_{i}^{B}e^{-t/10}$ perturbs the trajectory toward one of the two fixed points. Trajectories correspond to 60 time units.

The network of Fig. 7E multi-tasks with four limit cycle tasks, four feedforward sequences, and four fixed-point tasks in a network of 6000 rectified-linear neurons. The threshold θ=-0.3, and $g_{I}=1.6$. Each task is associated with a non-binary barcode (see Appendix A.8.3). A fraction of exactly f=0.3 of barcode entries are set to one. The other entries are zero for the $n^{\mu }$ patterns and δ=0.1 for the $m^{\mu }$ patterns. The limit cycle parameters $\left(\eta_{\mu },\omega_{\mu }\right)$ are (1.5, 0.6), (1.55, 1.0), (1.6, 1.5), and (1.65, 1.8). For the sequential tasks (Eq. 4), the number of dimensions $R_{\mu }$ per task and the feedforward gain $\left(R_{\mu },g_{\mu }\right)$ are (8, 1.4), (10, 1.45), (12, 1.5), and (14, 1.55). For the fixed-point tasks, strengths $\sigma_{\mu }$ are 1.5, 1.6, 1.65, and 1.7. Tasks are selected individually or in pairs in epochs of length 21 time units by applying an input of amplitude s=0.6 to neurons in the union of the selected barcodes. When a sequence is selected, the network receives an input proportional to the first pattern, divided by $\sqrt{2}$ when two sequences are selected simultaneously.

## Supplementary Material

1

## Figures and Tables

**Figure 1:**
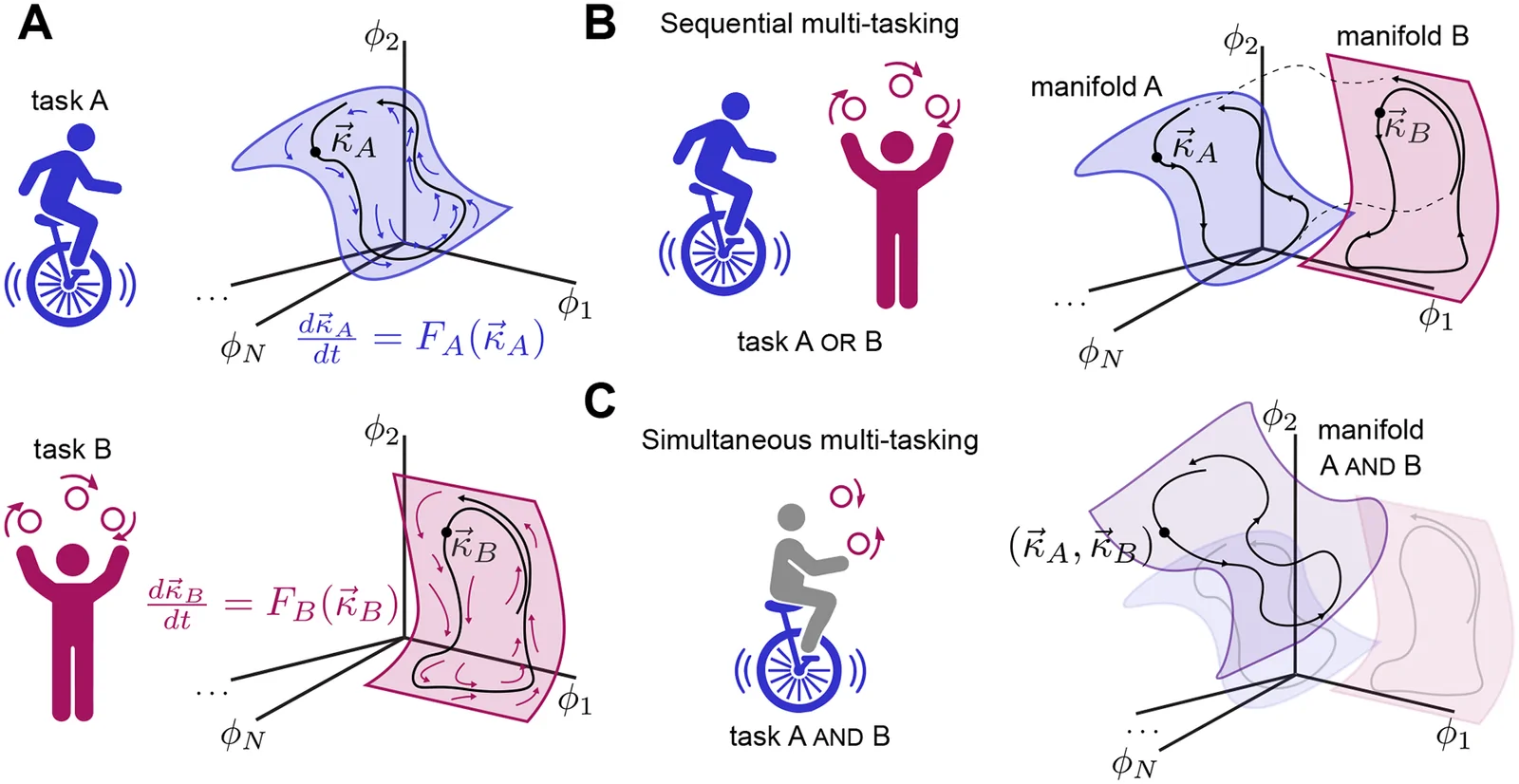
Task manifolds and multi-tasking. **A** Tasks, like cycling or juggling (left) are supported by computations that depend on dynamical interactions between task variables, denoted ${\vec{\kappa }}_{A}$ or ${\vec{\kappa }}_{B}$ for tasks A and B. These variables may be represented by neural activity that lies on a manifold in firing rate space (right; $\phi_{i}$ denotes firing rate of neuron i) which is parameterized by ${\vec{\kappa }}_{A}$ or ${\vec{\kappa }}_{B}$. The task variables evolve according to dynamics $F_{A}\left({\vec{\kappa }}_{A}\right)$ and $F_{B}\left({\vec{\kappa }}_{B}\right)$ that correspond to flow fields on each manifold (colored arrows). **B** Sequential multi-tasking requires performing one task at a time (left). This may be supported by neural activity that transitions between manifolds associated with each task and is thus parameterized by one set of task variables at a time (right). Parts of trajectories associated with transitions between manifolds are shown with dashed lines. **C** Simultaneous multi-tasking involves performance of multiple tasks at the same time (left). This may be supported by neural activity that is not confined to the manifold corresponding to any one task, but to a new, higher-dimensional manifold that combines them (purple region schematizes a 4-dimensional manifold). For two tasks A and B, the manifold is thus parameterized by $\left({\vec{\kappa }}_{A},{\vec{\kappa }}_{B}\right)$.

**Figure 2:**
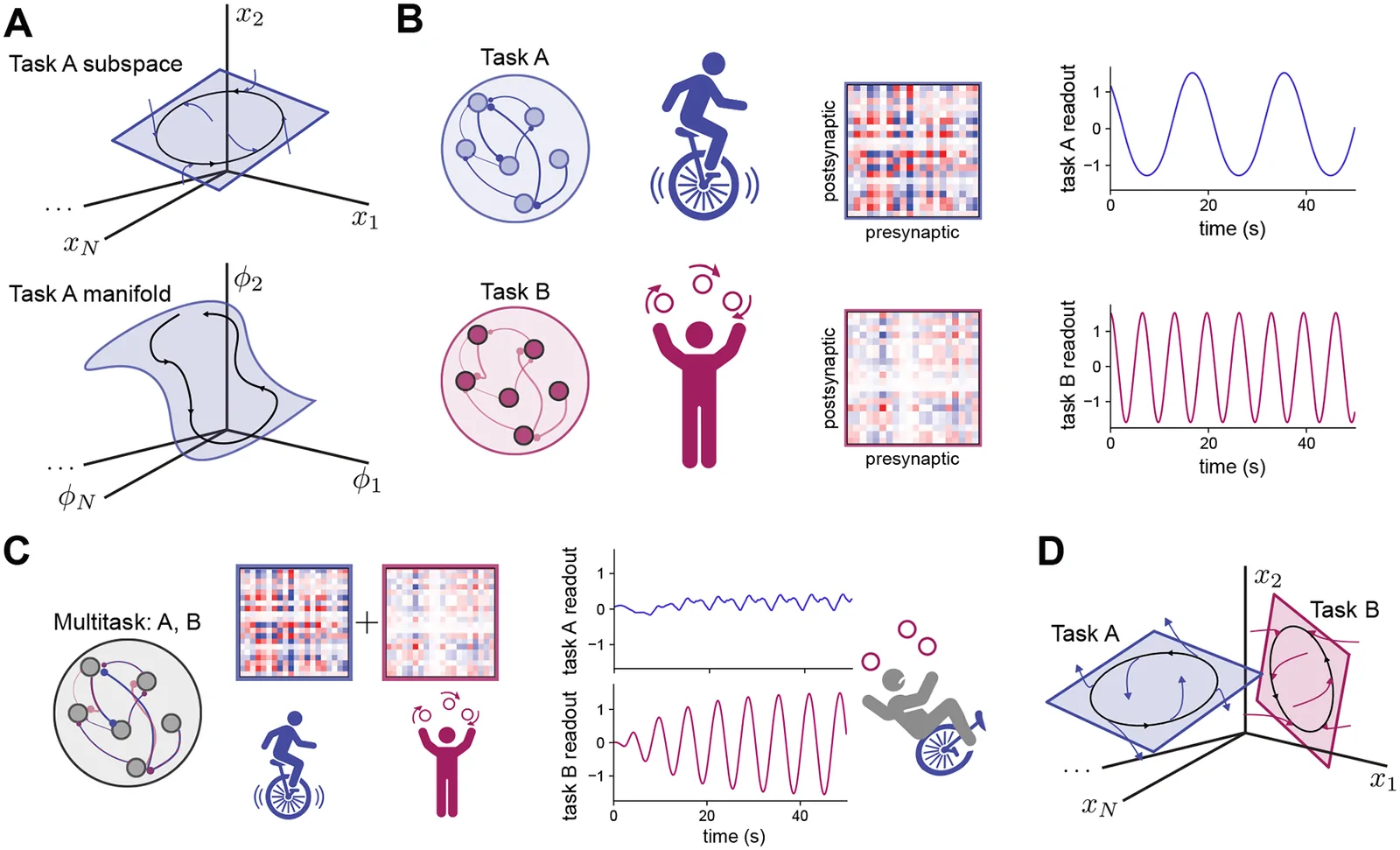
Task interference. **A** Illustration of a two-dimensional task subspace in the space of input currents $x_{i},i=1\ldots N$ (top) and a two-dimensional task manifold in the space of firing rates $\phi_{i}$ (bottom). Blue arrows in top panel indicate the subspace’s transverse stability: trajectories that are perturbed off the subspace are attracted to it. **B** Two networks with rank-two synaptic weights each produce oscillations with different frequencies (right). These oscillations model the computation each network performs in each of two different tasks, in this case cycling (top) or juggling (bottom). **C** A rank-four network whose synaptic weights are the sum of those in **B** (left) fails to multi-task: the task A output is suppressed by task B and occurs at an incorrect frequency (right). **D** Illustration of transverse instability of the task A subspace. Trajectories that are perturbed off the subspace are repelled from it and attracted to the task B subspace.

**Figure 3:**
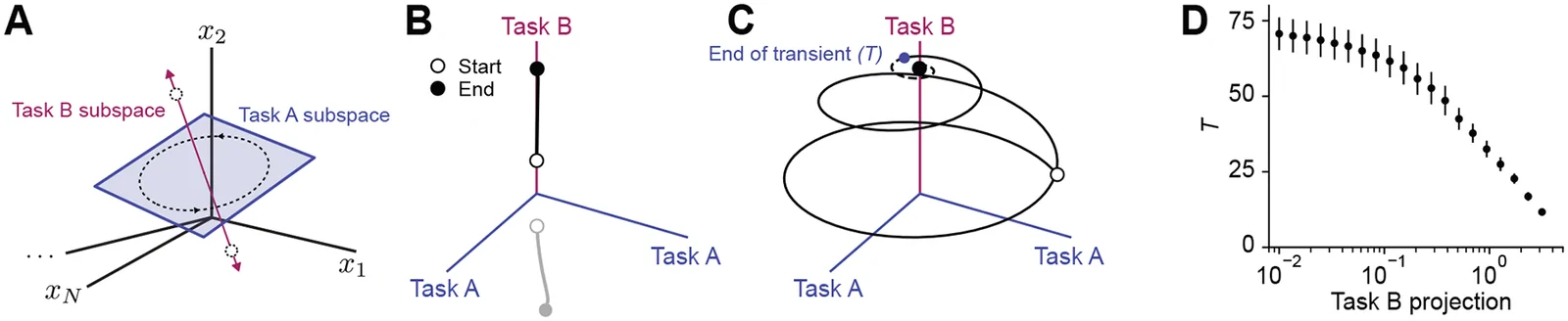
Sequential multi-tasking through transient activity. **A** A two-dimensional task A subspace is associated with a limit cycle (dashed ellipse) and a one-dimensional task B subspace is associated with bistable fixed points (dashed circles). **B** Two neural trajectories (black and gray) projected onto the three-dimensional space defined by the subspaces in **A**. Each initial condition (open circles) lies near the task B subspace and produces a trajectory that approaches one of the two fixed points by the end of the simulation (filled circles). **C** Similar to **B** but for a neural trajectory with initial condition near the task A subspace. The trajectory transiently oscillates before decaying to a fixed point in the task B subspace. Blue mark indicates the time point T at which the norm of the projection onto the task A subspace decays below r=0.25 times its initial value. Trajectory after this timepoint is illustrated with a dashed line. **D** Length of transient T as defined in **C**, in units of membrane time constant, as a function of the initial condition’s projection onto the task B subspace (relative to that for task A). Mean ± s.e.m. over 20 networks with N=10,000.

**Figure 4:**
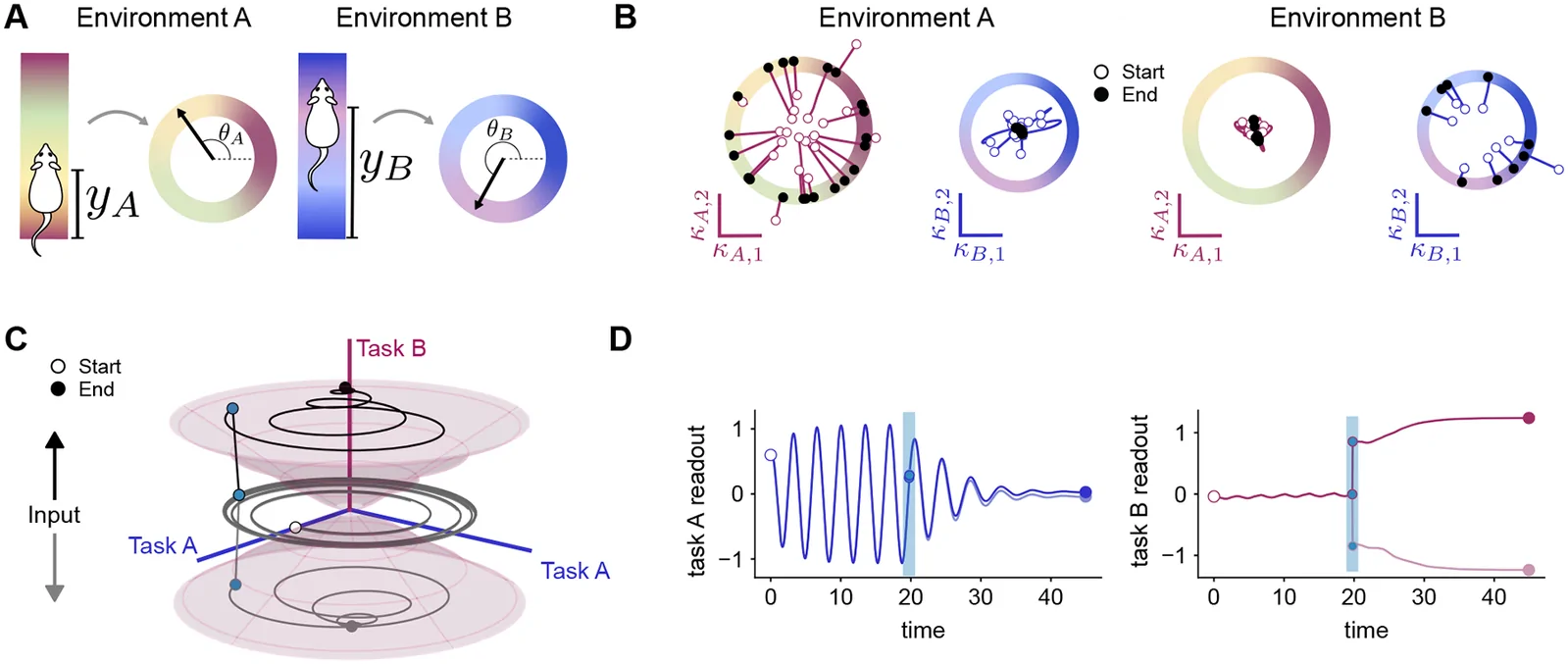
Multistability for two examples of sequential multi-tasking: **A-B** a model of place cells in two one-dimensional environments; **C-D** and a multi-stable combination of a limit cycle and bistable fixed points. **A** An animal represents its location $y_{A/B}$ in either of two one-dimensional environments (A or B) with a periodic encoding parameterized by $\theta_{A/B}$. **B** Dynamics of neural activity projected onto the subspace associated with each environment. Dynamics in each subspace feature a ring attractor. Locations on the attractor encode $\theta_{A/B}$. Left: Neural trajectories whose initial conditions (open circles) have high overlap with the environment A subspace converge toward a steady state on its corresponding ring (filled circles), while activity in the task B subspace decays. Right: The converse is true for initial conditions that have high overlap with the environment B subspace. **C** A multi-stable model of the limit cycle and memory tasks introduced in Fig. 3. In this model, elements of connectivity vectors follow statistics of a cosine bump, in contrast with Gaussian-distributed weights as in Fig. 3. Two neural trajectories projected onto the three-dimensional space defined as in Fig. 3. The initial condition first selects task A, then an input pulse positively (black) or negatively (gray) aligned with the task B subspace moves the trajectory into task B’s basin of attraction. Conical surfaces denote the separatrix between the task A and B basins of attraction. **D** Readouts of the task A and B subspaces as a function of time, for the trajectories in **C**. Blue vertical line indicates when the input pulse is provided.

**Figure 5:**
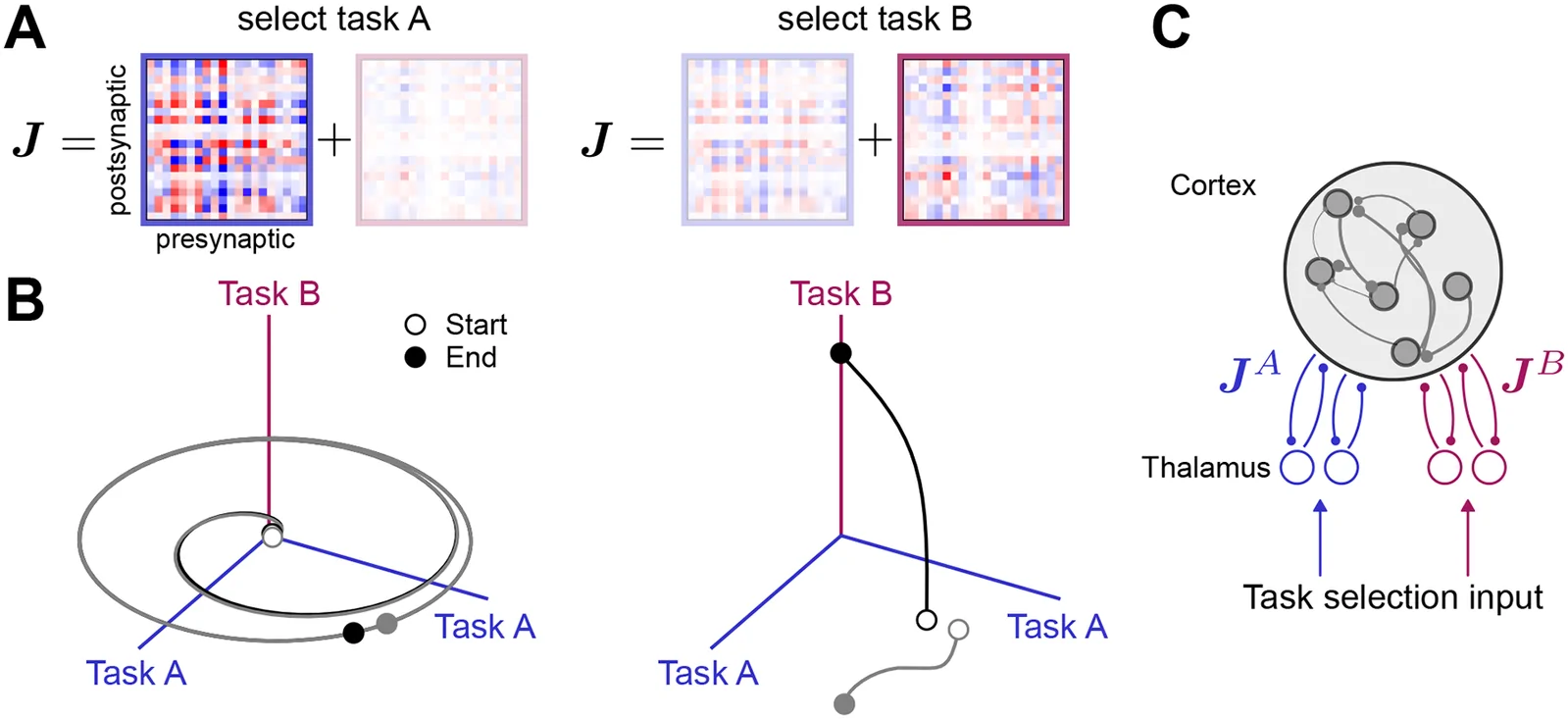
Sequential multi-tasking through changes in effective connectivity. **A** A model of the limit cycle and memory tasks with the same connectivity as in Fig. 3 and an additional factor that increases the magnitude of the task A or B connectivity component when the respective task is selected. **B** Two example neural trajectories (gray and black) over sequential selection of task A (left) and B (right). In contrast to Fig. 3, initial conditions are not precisely aligned to each subspace. Initial conditions for the right plot are endpoints of trajectories for the left plot. When task B is selected, an input, representing a sensory cue and either positively (black) or negatively (gray) aligned with the task B subspace, is briefly provided to perturb the neural trajectories toward different fixed points. **C** Anatomical basis for modulation of effective connectivity proposed by Logiaco et al.^15^. Connectivity components are associated with distinct corticothalamic loops.

**Figure 6:**
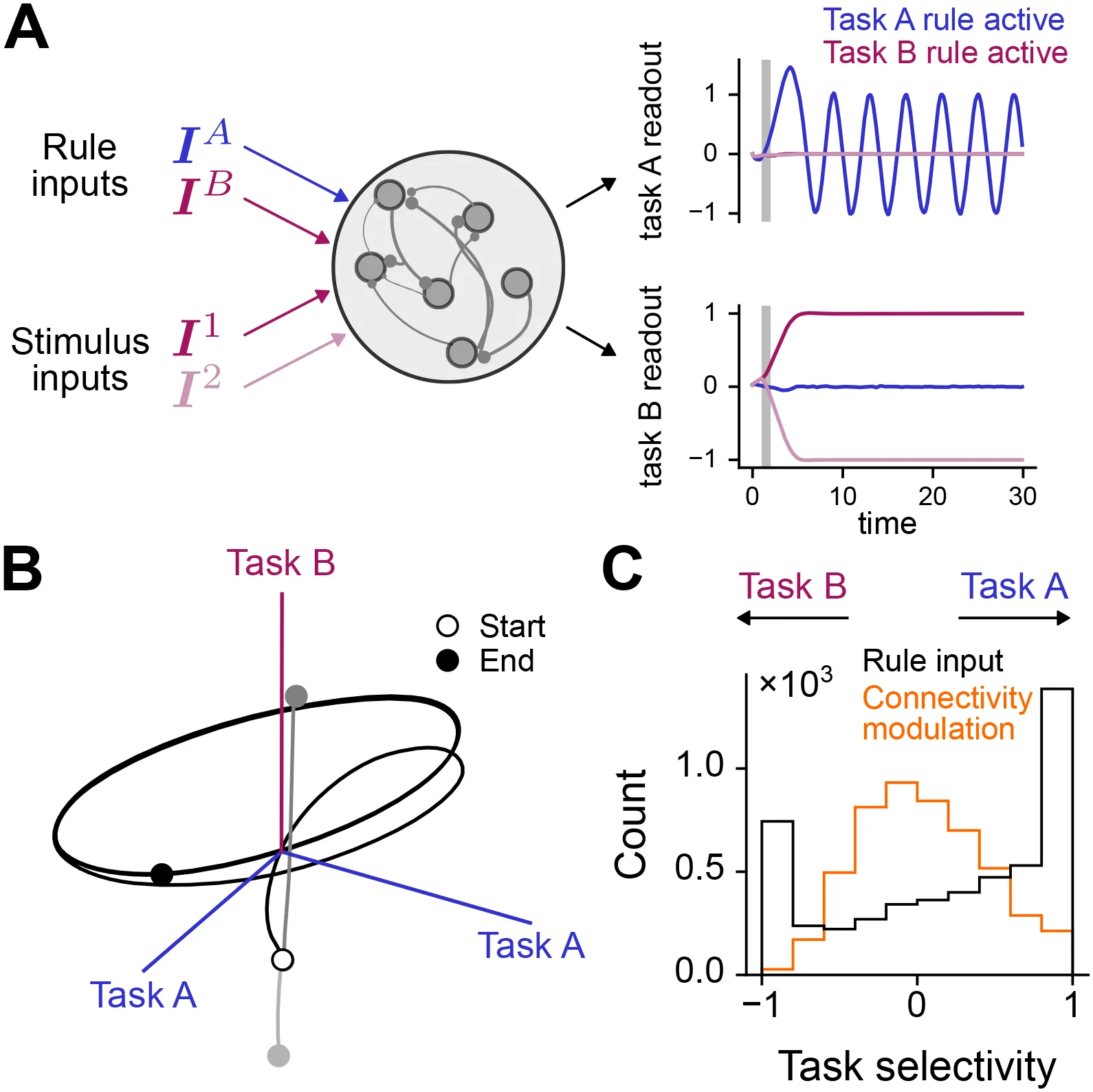
Sequential task selection by a rule input, in a full-rank network trained on the limit cycle and memory tasks introduced in Fig. 3. **A** Schematic illustrating a network receiving rule inputs to select task A or B and stimulus inputs to select the task B fixed point. Right: Linear readouts of a network trained to produce an oscillation when the task A rule input is active and bistable fixed points when the task B rule input is active. Loss is determined by readout activity for t>5. When task B is selected, a stimulus input indicating the correct task B fixed point is provided during the time interval indicated by the gray box. **B** Example neural trajectories with the same initial condition during selection by task A (black) or B (gray) rule inputs. Axes are defined by projections onto the top two principal components of task A trials and the top principal component of task B trials. Light and dark gray trajectories represent trials with distinct stimulus inputs. **C** Distribution of neuronal task selectivity index $\left(\sigma_{A}-\sigma_{B}\right)/\left(\sigma_{A}+\sigma_{B}\right)$, where $\sigma_{\mu }$ is the standard deviation of a neuron’s activity during the performance of task μ. Distribution is computed across neurons of 10 trained networks, each with N=500 (black), and compared to the distribution for networks that multi-task through modulation of effective connectivity as described in Fig. 5 (orange).

**Figure 7:**
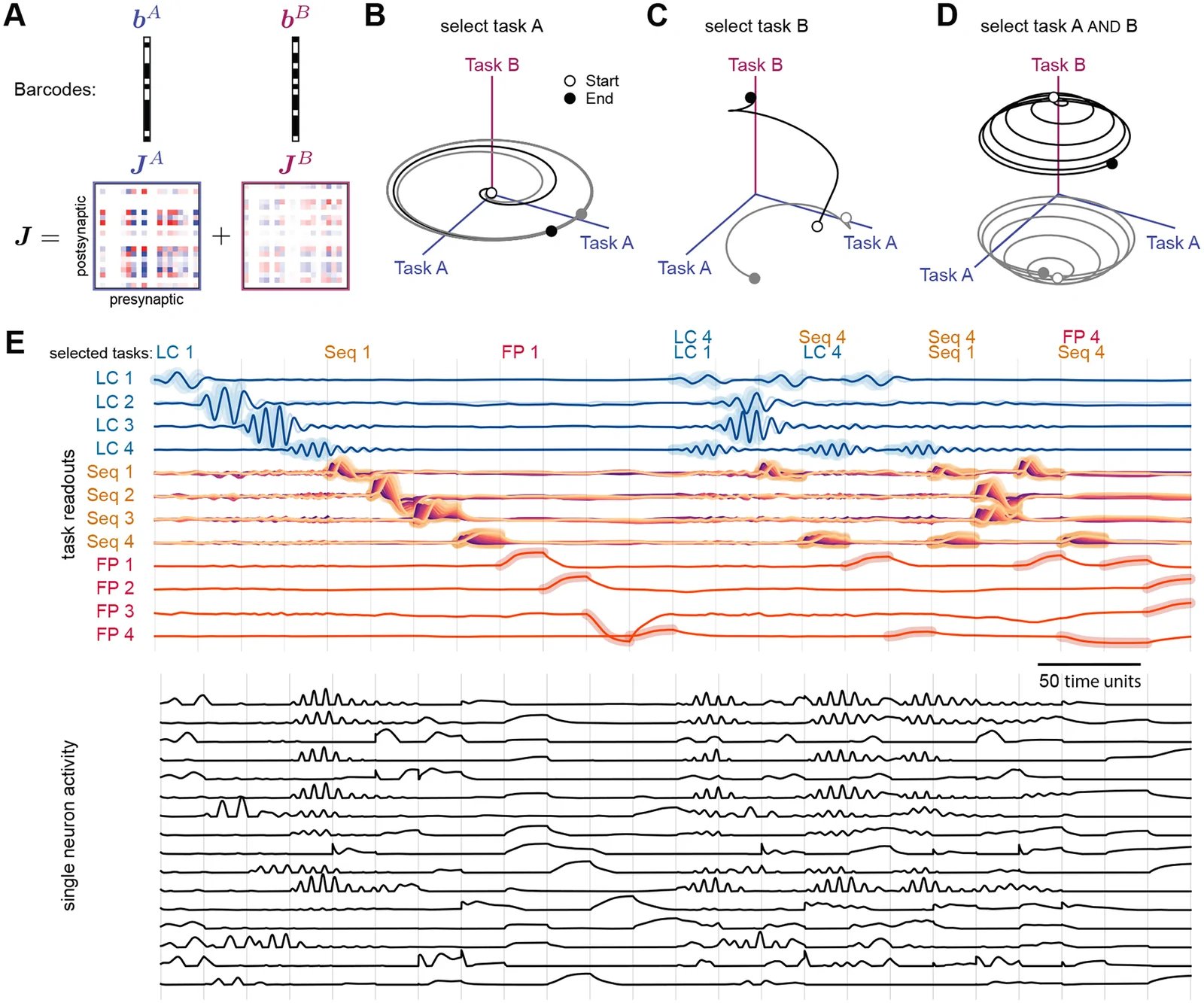
Barcoded connectivity. **A** Schematic of barcodes $b^{\mu }$ (top; black squares indicate positive values) and corresponding connectivity components $J^{\mu }$ (bottom). **B** Two example neural trajectories (gray and black) during selection of task A by a rule input. **C** Similar to **B** but during selection of task B. As in Fig. 5B, an input positively (black) or negatively (gray) aligned with the task B subspace is provided when task B is selected. **D** Similar to **C** but during simultaneous selection of both tasks by a rule input aligned with both barcodes. **E** Example network that performs 12 tasks: four limit cycles (LC), four sequences (Seq) and four fixed point attractors (FP). Different combinations of tasks are selected in each time epoch. Gray lines denote epoch boundaries. Top: Linear readouts of the different task manifolds. The highlighted background indicates when the corresponding task is selected by the rule input (labels at top of panel also indicate the selected task; not all epochs are labeled). For the first 12 epochs, only single tasks are selected, while for the last 12 epochs, two tasks are simultaneously selected. Bottom: Firing rates of 16 example neurons. Barcode sparsity parameter f=0.3.

**Table 1:** Comparison of multi-tasking mechanisms.

Task selection mechanism	Simultaneous or sequential	Dynamical picture	Control parameter	References
Preparatory activity/transients	Sequential	One flow field, trajectory determined by initial state	Precision of initial state	13, 35
Multistability	Sequential	One flow field with multiple attractors, trajectory determined by initial state	Distribution of task selectivities	29, 36–38
Change in effective connectivity	Sequential	Flow field changes as connectivity changes	Magnitude of connectivity modulation $\left(a_{\mu }\right)$	15, 18, 30, 43
Rule input	Both	Flow field changes with rule input	Degree of modularity (barcode sparsity f)	9, 10
