# Supplementary material for: Neural circuit mechanisms for multi-tasking
Source: bioRxiv. 2026 Oct 7:2026.10.01.755960. Preprint. [Version 1] doi: 10.64898/2026.10.01.755960 (PMC13691935; doi:10.64898/2026.10.01.755960)
Supplement: Supplementary file 1 [file NIHPP2026.10.01.755960V1-supplement-1.pdf]

bioRxiv preprint doi: https://doi.org/10.64898/2026.10.01.755960; this version posted October 8, 2026. The copyright holder for this preprint (which was not certified by peer review) is the author/funder, who has granted bioRxiv a license to display the preprint in perpetuity. It is made available under a CC-BY 4.0 International license.

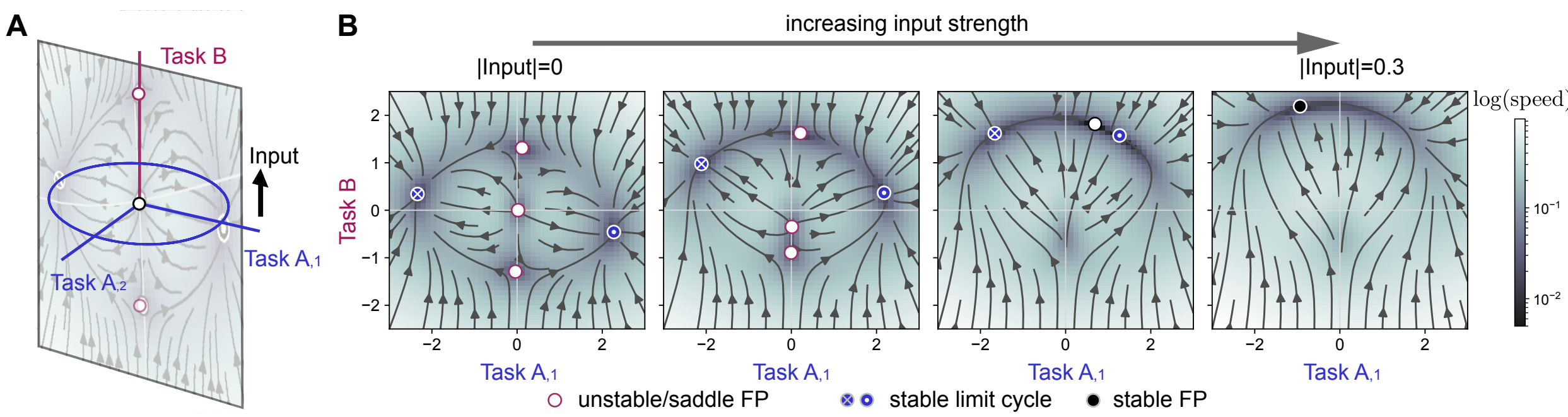

Figure S1: External inputs with Gaussian statistics do not affect relative transverse instability.
A Illustration of a rank-three network with Gaussian connectivity statistics, performing task A (a limit cycle, blue line) and task B (bistable fixed point). Task B is unstable when perturbed along the Task A dimension. We add a tonic Gaussian input along the Task B axis (black line, input parallel to $\boldsymbol{m}^B$). We focus on the dynamics on the illustrated two-dimensional slice.
B Flow field along the two-dimensional plane defined by the task-B variable and one of the two task-A variables. The different panels show increasing input strength (0, 0.1, 0.2 and 0.3). The input along Task B cannot stabilize Task B. As the input increases, the network undergoes a first bifurcation, where the bistable fixed points along the task-B manifold turn into a single input-driven fixed point, which remains transversely unstable. A second bifurcation happens when the stable limit cycle collapses into an input-driven stable fixed point. The recurrent dynamics associated with task B are never stable, independently of the external input. Network size $N = 10{,}000$.

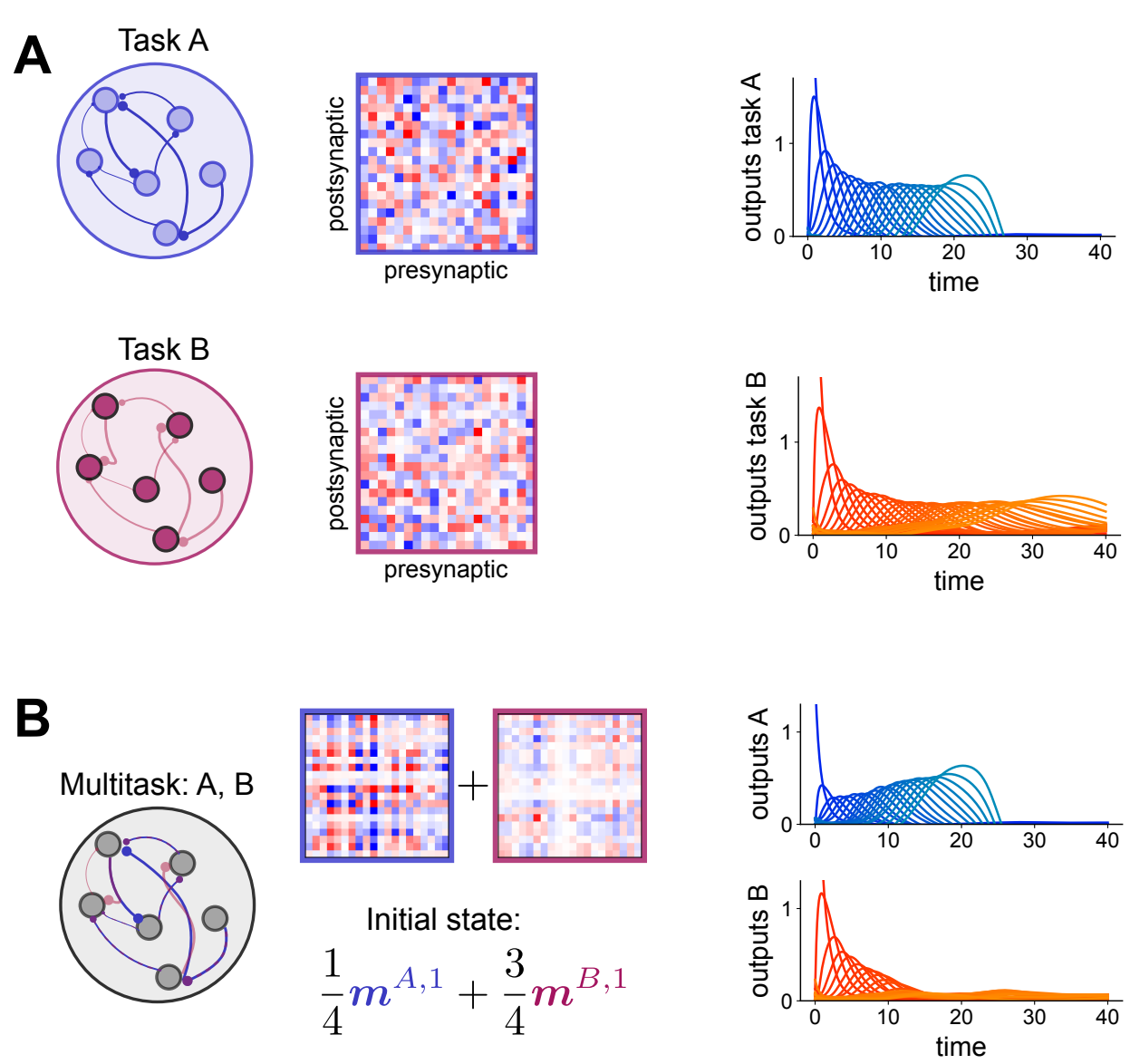

Figure S2: Interference between sequences.
A Task A consists of a sequence of 20 different patterns, Task B consists of a sequence of 30 different patterns. Individual networks can reproduce such sequence of activity, with a sequence duration approximately equal to the number of patterns. The connectivity follows Eq. (4) [13].
B When the connectivity matrices corresponding to both tasks are combined into a single network, the task with the strongest weights (task A in this example) is stable, while task B becomes transversely unstable. When initialized with an initial state overlapping with both sequences, only task A can be executed until the end, even if task B has a stronger initial overlap.

bioRxiv preprint doi: https://doi.org/10.64898/2026.10.01.755960; this version posted October 8, 2026. The copyright holder for this preprint (which was not certified by peer review) is the author/funder, who has granted bioRxiv a license to display the preprint in perpetuity. It is made available under a CC-BY 4.0 International license.

Appendix: Supplemental theoretical results

A.1 Definition of multi-tasking manifolds

Consider a set of tasks indexed by $\mu = 1 \dots P$, each defined by dynamics F_μ on its task variables $\boldsymbol{\kappa}_\mu$:

$$\dot{\boldsymbol{\kappa}}_\mu = F_\mu(\boldsymbol{\kappa}_\mu). \tag{1}$$

The vector $\boldsymbol{\kappa}_\mu$ has R_μ elements and represents quantities relevant for performance of task μ.

We now consider a recurrent neural network and define what it means for the dynamical system governing its activity to have manifolds that permit sequential or simultaneous multi-tasking of tasks defined by Eq. (1). Suppose there is an invariant manifold in the network's activity space, and locations on the manifold are smoothly parameterized by $\boldsymbol{z}$. Denote the dynamics of $\boldsymbol{z}$ on this manifold by $\dot{\boldsymbol{z}} = G(\boldsymbol{z})$. The function G is determined by the dynamical system that governs neural activity, evaluated at locations on the manifold.

We next need a notion of what it means for the dynamics of $\boldsymbol{z}$ to be equivalent to those of the task variables (Eq. 1). We use a property called *topological conjugacy*, which, informally, requires existence of a continuous, invertible transformation that maps trajectories of one dynamical system to another while preserving time. This notion has been previously used to assess similarity of dynamics in neural activity space[69]. Conjugate dynamical systems have the same global structure, such as their arrangements of fixed points and limit cycles. Formally, if two dynamical systems are defined by[†] $\boldsymbol{z}(t+T) = \phi_T(\boldsymbol{z}(t))$ and $\boldsymbol{\kappa}(t+T) = \psi_T(\boldsymbol{\kappa}(t))$, they are topologically conjugate if there is a homeomorphism $\boldsymbol{\kappa} = h(\boldsymbol{z})$ such that $h \circ \phi = \psi \circ h$. This notion of equivalence is intentionally broad, and for several of the examples we consider h is the identity function.

With this definition, we say that a system *supports sequential multi-tasking of tasks μ and ν* if it has a distinct attracting manifold for each task and the dynamics on these manifolds are conjugate to those defined by F_μ and F_ν, respectively. The dimensions of these manifolds are therefore R_μ and R_ν.

To define dynamics associated with performing tasks μ and ν simultaneously, let $\boldsymbol{\kappa} = (\boldsymbol{\kappa}_\mu, \boldsymbol{\kappa}_\nu)$ with dynamics given by $\dot{\boldsymbol{\kappa}} = (F_\mu(\boldsymbol{\kappa}_\mu), F_\nu(\boldsymbol{\kappa}_\nu)) \equiv F_{\mu\nu}(\boldsymbol{\kappa})$. This dynamical system corresponds to two uncoupled sets of task variables, each following Eq. (1). A system *supports simultaneous multi-tasking of tasks μ and ν* if it has an attracting manifold and the dynamics on this manifold are conjugate to those defined by $F_{\mu\nu}$. The dimension of this manifold is therefore $R_\mu + R_\nu$. This definition can be generalized to more than two tasks by defining $\boldsymbol{\kappa}$ in terms of each task's variables.

In some cases, there may be neural activity dynamics unrelated to the task variables, leading to higher-dimensional manifolds. In this case, the definitions can be extended by defining the dynamics in terms of $\boldsymbol{\kappa} = (\boldsymbol{\kappa}_\mu, \boldsymbol{\kappa}_0)$ or $\boldsymbol{\kappa} = (\boldsymbol{\kappa}_\mu, \boldsymbol{\kappa}_\nu, \boldsymbol{\kappa}_0)$ for sequential and simultaneous multi-tasking, respectively, where $\boldsymbol{\kappa}_0$ describes these unrelated dynamics.

To show that a manifold satisfies one of these definitions, it is necessary to show both that it is attracting and that the dynamics on it are conjugate to those of the appropriate task variables. The networks we construct that perform transient multi-tasking (Fig. 3) satisfy the conjugacy requirement but not the attracting requirement. We note that, depending on which values of $\boldsymbol{\kappa}_\mu$

[†]In terms of G and F_μ, for example, $\phi_T(\boldsymbol{z}(t)) = \boldsymbol{z}(t) + \int_t^{t+T} ds\, G(\boldsymbol{z}(s))$ and $\psi_T(\boldsymbol{\kappa}_\mu(t)) = \boldsymbol{\kappa}_\mu(t) + \int_t^{t+T} ds\, F_\mu(\boldsymbol{\kappa}_\mu(s))$.

bioRxiv preprint doi: https://doi.org/10.64898/2026.10.01.755960; this version posted October 8, 2026. The copyright holder for this preprint (which was not certified by peer review) is the author/funder, who has granted bioRxiv a license to display the preprint in perpetuity. It is made available under a CC-BY 4.0 International license.

are relevant for task performance, one might choose to require these properties hold for only a subset of task variable values. For example, if $\boldsymbol{\kappa}_\mu$ represents a set of joint angles, the behavior of Eq. (1) for unachievable joint configurations is unimportant. We now investigate these properties for specific low-rank recurrent neural network models.

A.2 Task variable dynamics in low-rank recurrent neural networks

We assume a network of the form

$$J_{ij} = -g_I/N + \sum_{\mu=1}^{P} a_\mu J_{ij}^\mu, \qquad J_{ij}^\mu = \frac{1}{N}\sum_{r=1}^{R_\mu} m_i^{r,\mu} n_j^{r,\mu}. \tag{2}$$

Except for the case of modulation of effective connectivity, (Section A.6.4), we assume $a_\mu = 1$ for all μ. Activity in the network can be parameterized using a set of latent variables $\kappa_{\mu,r}(t)$, along with a variable $\bar{x}(t)$ representing mean activity:

$$\boldsymbol{x}(t) = \sum_{\mu=1}^{P}\sum_{r=1}^{R_\mu} \boldsymbol{m}^{\mu,r}\kappa_{\mu,r}(t) + \mathbf{1}\bar{x}(t). \tag{3}$$

Activity decays along all directions except the span of $\{\boldsymbol{m}^{\mu,r}\}$ and $\mathbf{1}$. For now, we further assume that there is no global inhibition, $g_I = 0$, such that $\bar{x}$ approaches 0. Later we will discuss how inhibition, and therefore time-varying values of $\bar{x}$, affects the dynamics (Sec. A.9). An advantage of considering low-rank recurrent neural networks is that, in terms of $\boldsymbol{x}(t)$, task-related activity corresponds to a linear subspace rather than a nonlinear manifold (although the nonlinearity $\phi(\boldsymbol{x})$ produces a nonlinear manifold in firing rate space). We define the *task subspace* associated with task μ as the subspace of $\boldsymbol{x}$ in which $\boldsymbol{\kappa}_\mu$ takes any value, but $\boldsymbol{\kappa}_\nu = \mathbf{0}$ for all $\nu \neq \mu$.

The starting point for our analysis is the assumption that single connectivity components alone are sufficient to perform single tasks. That is, if $J_{ij} = J_{ij}^\mu$, the variables $\boldsymbol{\kappa}_\mu$ that define the neural activity state (Eq. 3) follow the desired dynamics $\dot{\boldsymbol{\kappa}}_\mu = F_\mu(\boldsymbol{\kappa}_\mu)$. This assumption is based on extensive prior literature on low-rank recurrent neural networks as models of single-task computation[16,19–21].

To simplify notation, we will describe most results in the setting of networks that perform two tasks $\mu \in \{A, B\}$, but generalizations to more tasks will be noted. The dynamics are

$$\dot{\boldsymbol{\kappa}}_A = G_A(\boldsymbol{\kappa}_A, \boldsymbol{\kappa}_B), \tag{4}$$

$$\dot{\boldsymbol{\kappa}}_B = G_B(\boldsymbol{\kappa}_A, \boldsymbol{\kappa}_B). \tag{5}$$

The key issue is that G_A and G_B, in general, depend on both $\boldsymbol{\kappa}_A$ and $\boldsymbol{\kappa}_B$ and are thus different from F_A and F_B. We refer to this coupling of task variables across different tasks as "interactions." The form of these interactions determines whether a network with summed connectivity components satisfies the criteria for multi-tasking.

A.3 Readouts of task-related activity

Our criteria for multi-tasking focus on dynamics on task manifolds without requiring the form of the readout of this activity to be specified. The simplest interpretation associates to each task a distinct linear readout y^μ, defined by weights $\boldsymbol{w}^\mu$ that have nonzero alignment with the μth task manifold, so that $y^\mu(t) = \boldsymbol{w}^\mu \cdot \phi(\boldsymbol{x}(t))$. Alternatively a shared readout $y(t)$ may be generated through a single weight vector given by $\sum_\mu \boldsymbol{w}^\mu$, so that $y(t) = (\sum_\mu \boldsymbol{w}^\mu) \cdot \phi(\boldsymbol{x}(t))$.

bioRxiv preprint doi: https://doi.org/10.64898/2026.10.01.755960; this version posted October 8, 2026. The copyright holder for this preprint (which was not certified by peer review) is the author/funder, who has granted bioRxiv a license to display the preprint in perpetuity. It is made available under a CC-BY 4.0 International license.

It is also possible to modify the synaptic weight matrix, Eq. (2), so that readout-related activity is represented by the projection along a distinct subspace in neural activity space. Specifically, suppose

$$J_{ij}^{\mu} = \frac{1}{N}\sum_{r=1}^{R_\mu} m_i^{r,\mu} n_j^{r,\mu} + \frac{1}{N} m_i^{\text{out}} n_j^{\text{out},\mu}, \tag{6}$$

where the elements of $\boldsymbol{m}^{\text{out}}$ are random and uncorrelated with all other connectivity vectors, and $\boldsymbol{n}^{\text{out},\mu}$ is a vector in the μth task subspace along which readout-related activity evolves. This creates an effectively feed-forward projection of neural activity along $\boldsymbol{m}^{\text{out}}$, which forms a readout that is shared across tasks but only weakly feeds back into the task-related dynamics. These definitions can be straightforwardly generalized to multiple readout dimensions.

A.4 Transverse stability

We require that a task manifold be attracting, meaning that nearby trajectories must return to it. A related requirement is what we will call *local transverse stability*, which is determined by the stability of the linearized dynamics orthogonal to the manifold and evaluated at a fixed location on the manifold. Local transverse stability of the μth task subspace in networks of the form of Eq. (2) is determined by deviations in $\boldsymbol{\kappa}_\nu$, where $\nu \neq \mu$, away from $\boldsymbol{\kappa}_\nu = \boldsymbol{0}$. These deviations can be characterized by the transverse Jacobian

$$\boldsymbol{T}_{\mu\nu}(\boldsymbol{\kappa}_\mu) \equiv \nabla_{\boldsymbol{\kappa}_\nu} G_\nu(\boldsymbol{\kappa}_\mu, \boldsymbol{0}), \tag{7}$$

where $\dot{\boldsymbol{\kappa}}_\nu = G_\nu(\boldsymbol{\kappa}_\mu, \boldsymbol{0})$ governs the dynamics of $\boldsymbol{\kappa}_\nu$ when $\boldsymbol{\kappa}_\tau = \boldsymbol{0}$ for all $\tau \neq \mu$. $\boldsymbol{T}_{\mu\nu}(\boldsymbol{\kappa}_\mu)$ is thus interpreted as the linearization of the transverse dynamics of $\boldsymbol{\kappa}_\nu$, evaluated at a point $\boldsymbol{\kappa}_\mu$ in the task μ subspace. Because each subspace is orthogonal, local transverse stability can be evaluated by requiring, for each $\nu \neq \mu$ separately, that all eigenvalues of $\boldsymbol{T}_{\mu\nu}(\boldsymbol{\kappa}_\mu)$ have negative real part.

Local transverse stability everywhere on a manifold does not always guarantee it is attracting[†]. However, it will be sufficient in our models because the transverse eigenvectors are the same at each point in a task subspace. If the associated eigenvalues always have negative real parts, projections onto these eigenvectors will decay.

A.5 Simultaneous multi-tasking in linear networks with orthogonal subspaces

We first consider networks where the single-neuron transfer function is $\phi(x) = x$. In this case, defining $(\boldsymbol{\sigma}_{\mu\nu})_{rr'} = \frac{1}{N}\sum_{i=1}^{N} n_i^{\mu,r} m_i^{\nu,r'}$, Eqs. (4) and (5) become:

$$G_A(\boldsymbol{\kappa}_A, \boldsymbol{\kappa}_B) = -\boldsymbol{\kappa}_A + \boldsymbol{\sigma}_{AA}\boldsymbol{\kappa}_A + \boldsymbol{\sigma}_{AB}\boldsymbol{\kappa}_B, \tag{8}$$

$$G_B(\boldsymbol{\kappa}_A, \boldsymbol{\kappa}_B) = -\boldsymbol{\kappa}_B + \boldsymbol{\sigma}_{BA}\boldsymbol{\kappa}_A + \boldsymbol{\sigma}_{BB}\boldsymbol{\kappa}_B. \tag{9}$$

In this case, there are no interactions between tasks if connectivity vectors corresponding to different tasks are mutually orthogonal: $\boldsymbol{m}^\mu \perp \boldsymbol{m}^\nu$, $\boldsymbol{n}^\mu \perp \boldsymbol{n}^\nu$, $\boldsymbol{m}^\mu \perp \boldsymbol{n}^\nu$, for $\mu \neq \nu$. When the last of these

[†]Counterexamples can be constructed by considering the system $\dot{\boldsymbol{x}} = \boldsymbol{A}(t)\boldsymbol{x}$, and interpreting t as the location along a one-dimensional manifold and $\boldsymbol{x}$ as the transverse dynamics. Josić & Rosenbaum[75] provide a didactic account of several counterintuitive examples in which $\boldsymbol{A}(t)$ has eigenvalues with negative real part for all t, yet $\boldsymbol{x} = \boldsymbol{0}$ is unstable. The examples rely on the eigenvectors of $\boldsymbol{A}(t)$ changing with t.

bioRxiv preprint doi: https://doi.org/10.64898/2026.10.01.755960; this version posted October 8, 2026. The copyright holder for this preprint (which was not certified by peer review) is the author/funder, who has granted bioRxiv a license to display the preprint in perpetuity. It is made available under a CC-BY 4.0 International license.

requirements holds, $\boldsymbol{\sigma}_{AB} = \boldsymbol{\sigma}_{BA} = 0$, so that the dynamics of $\boldsymbol{\kappa}_A$ and $\boldsymbol{\kappa}_B$ decouple into $\dot{\boldsymbol{\kappa}}_A = F_A(\boldsymbol{\kappa}_A)$, $\dot{\boldsymbol{\kappa}}_B = F_B(\boldsymbol{\kappa}_B)$. Because activity in directions orthogonal to the task A and B subspaces decays, this satisfies the criteria for simultaneous multi-tasking, with the limitation that single tasks must correspond to linear dynamical systems.

A.6 Multi-tasking in nonlinear networks: mean-field approach

We now consider nonlinear networks in the large-N, mean-field limit. We will again assume that the connectivity vectors for different tasks are orthogonal. This assumption is, in general, stronger than required for topological conjugacy, since some degree of overlap across tasks may distort but not completely reconfigure task-associated flow fields. The amount of overlap that can be tolerated will depend on the specifics of the tasks and the network.

The more significant observation for our present analysis is that, even when the connectivity vectors are orthogonal, the criteria for multi-tasking may not be satisfied in nonlinear networks. In particular, we show that while the dynamics in each task subspace may correctly correspond to those of the desired task, these subspaces may not be attracting.

A.6.1 Orthogonal and independent connectivity vectors preserve dynamics within task subspaces

The mean field limit we consider corresponds to assuming that, for each neuron i, the components of its connectivity vectors are drawn from a probability distribution, $\{m_i^{\mu,r}, n_i^{\nu,r'}\} \sim p(\{m^{\mu,r}, n^{\nu,r'}\})$, independently across neurons, for $\mu, \nu = 1 \dots P$ and $r, r' = 1 \dots R_{\mu/\nu}$. Under this assumption, sums of the form $\frac{1}{N}\sum_{i=1}^{N} f(\{m_i^{\mu,r}, n_i^{\nu,r'}\})$ converge to $\mathbb{E}[f(\{m^{\mu,r}, n^{\nu,r'}\})]$ where $\mathbb{E}$ is the expectation under the distribution p.

Beyond the mean-field assumption, we will further assume that connectivity components are statistically independent across tasks, so that $p(\{m^{\mu,r}, n^{\nu,r'}\}) = \prod_{\mu=1}^{P} p(\{m^{\mu,r}, n^{\mu,r'}\})$, and that each connectivity vector has zero-mean elements, $\mathbb{E}[m^{\mu,r}] = \mathbb{E}[n^{\mu,r}] = 0$. These two assumptions imply that connectivity vectors corresponding to different tasks are orthogonal in the large-N limit.

Under these assumptions, we first show that the dynamics restricted to the task A subspace are the same as F_A, the dynamics of a single-task network. Let $\boldsymbol{M}^\mu$ and $\boldsymbol{N}^\mu$ be $N \times R_\mu$ matrices whose rth columns are equal to $\boldsymbol{m}^{\mu,r}$ and $\boldsymbol{n}^{\mu,r}$, respectively. The dynamics of $\boldsymbol{\kappa}_A$ follow

$$G_A(\boldsymbol{\kappa}_A, \mathbf{0}) = -\boldsymbol{\kappa}_A + \frac{1}{N}(\boldsymbol{N}^A)^\top \phi(\boldsymbol{M}^A\boldsymbol{\kappa}_A + \boldsymbol{M}^B\mathbf{0}) \tag{10}$$

$$= -\boldsymbol{\kappa}_A + \frac{1}{N}(\boldsymbol{N}^A)^\top \phi(\boldsymbol{M}^A\boldsymbol{\kappa}_A) \tag{11}$$

$$= F_A(\boldsymbol{\kappa}_A). \tag{12}$$

Now we consider the dynamics of $\boldsymbol{\kappa}_B$. The fact that the connectivity components for tasks A and B are independent and zero-mean implies that $G_B = 0$, because

$$G_B(\boldsymbol{\kappa}_A, \mathbf{0}) = \frac{1}{N}(\boldsymbol{N}^B)^\top \phi(\boldsymbol{M}^A\boldsymbol{\kappa}_A), \tag{13}$$

bioRxiv preprint doi: https://doi.org/10.64898/2026.10.01.755960; this version posted October 8, 2026. The copyright holder for this preprint (which was not certified by peer review) is the author/funder, who has granted bioRxiv a license to display the preprint in perpetuity. It is made available under a CC-BY 4.0 International license.

and, for any r,

$$\frac{1}{N}(\boldsymbol{n}^{B,r})^\top \phi(\boldsymbol{M}^A \boldsymbol{\kappa}_A) = \mathbb{E}\left[n^{B,r}\phi\left(\sum_{r'} m^{A,r'}\kappa_{A,r'}(t)\right)\right] \tag{14}$$

$$= \mathbb{E}\left[n^{B,r}\right]\mathbb{E}\left[\phi\left(\sum_{r'} m^{A,r'}\kappa_{A,r'}(t)\right)\right] = 0. \tag{15}$$

This, along with Eq. (12), implies that, on the task A subspace, $\dot{\boldsymbol{\kappa}}_A = F_A(\boldsymbol{\kappa}_A)$ and $\dot{\boldsymbol{\kappa}}_B = \mathbf{0}$. An analogous argument holds for task B and for any finite number of tasks in which all pairs of connectivity components are orthogonal and independent. These dynamics clearly satisfy the topological conjugacy requirement for sequential multi-tasking, with the mapping from single-task to multi-task dynamics being the identity function.

A.6.2 Gaussian connectivity vectors lead to transverse instability

The next requirement for multi-tasking is that each task subspace is attracting. We show that, if $p(\{m^{\mu,r}, n^{\nu,r'}\})$, the probability distribution that defines the connectivity vectors, is jointly Gaussian, and tasks involve sustained dynamics, only one subspace satisfies this criterion, so that such networks do not successfully multi-task (as in Fig. 2C). It can be shown that, in this case, the dynamics of $\boldsymbol{\kappa}_\mu$ follow[16,21]:

$$\dot{\boldsymbol{\kappa}}_\mu = -\boldsymbol{\kappa}_\mu + g(\|\boldsymbol{\kappa}\|)\boldsymbol{\sigma}_\mu \boldsymbol{\kappa}_\mu, \tag{16}$$

where $(\boldsymbol{\sigma}_\mu)_{rr'} = \mathbb{E}[n^\mu_r m^\mu_{r'}]$, $\|\boldsymbol{\kappa}\|^2 = \sum_\nu \|\boldsymbol{\kappa}_\nu\|^2$ and $g = \mathbb{E}[\phi'(x)]$ is a scalar function, representing the average neural gain across the network. We assume ϕ is nondecreasing, so that $g \geq 0$. Eq. (16) implies that the transverse stability of task A with respect to task B is determined by the transverse Jacobian (Eq. 7):

$$\boldsymbol{T}_{AB}(\boldsymbol{\kappa}_A) = \nabla_{\boldsymbol{\kappa}_B} G_B(\boldsymbol{\kappa}_A, \mathbf{0}) = \nabla_{\boldsymbol{\kappa}_B}\left[-\boldsymbol{\kappa}_B + g(\|\boldsymbol{\kappa}\|)\boldsymbol{\sigma}_B\boldsymbol{\kappa}_B\right]_{(\boldsymbol{\kappa}_A,\mathbf{0})} \tag{17}$$

$$= -\boldsymbol{I} + g(\|\boldsymbol{\kappa}_A\|)\boldsymbol{\sigma}_B. \tag{18}$$

Note that the eigenvectors of Eq. (18) do not depend on $\boldsymbol{\kappa}_A$, which implies that local transverse stability determines whether the task A subspace is attracting, as discussed in Sec. A.4. Let $\alpha(\boldsymbol{\sigma})$ denote the largest real part of the eigenvalues of $\boldsymbol{\sigma}$. Assuming this quantity is positive, the largest real part of the transverse eigenvalues is then

$$\alpha(\boldsymbol{T}_{AB}(\boldsymbol{\kappa}_A)) = -1 + g(\|\boldsymbol{\kappa}_A\|)\alpha(\boldsymbol{\sigma}_B). \tag{19}$$

We now argue that, for the tasks we consider, if $\alpha(\boldsymbol{\sigma}_B) > \alpha(\boldsymbol{\sigma}_A)$, the task A subspace is locally transversely unstable. The argument relies on an assumption that, for every task, $g(\|\boldsymbol{\kappa}_\mu\|)\alpha(\boldsymbol{\sigma}_\mu) \geq 1$ for at least one value of $\boldsymbol{\kappa}_\mu$. In Sec. A.6.5, we show that this requirement is necessary for any task with sustained dynamics, i.e., for which $\boldsymbol{\kappa}(t)$ does not decay to 0 for large t.

However, since $\alpha(\boldsymbol{\sigma}_B) > \alpha(\boldsymbol{\sigma}_A)$, for any point at which $g(\|\boldsymbol{\kappa}_A\|)\alpha(\boldsymbol{\sigma}_A) \geq 1$, Eq. (19) is positive, and thus the task A subspace is locally transversely unstable. The same argument implies that if $\alpha(\boldsymbol{\sigma}_A) > \alpha(\boldsymbol{\sigma}_B)$, the task B subspace is locally transversely unstable. More generally, all task subspaces except for those with the largest value of $\alpha(\boldsymbol{\sigma}_\mu)$ are locally transversely unstable. Avoiding interference due to this instability would require fine-tuning to ensure all $\alpha(\boldsymbol{\sigma}_\mu)$ are equal. This limitation underlies the task interference shown in Fig. 2.

bioRxiv preprint doi: https://doi.org/10.64898/2026.10.01.755960; this version posted October 8, 2026. The copyright holder for this preprint (which was not certified by peer review) is the author/funder, who has granted bioRxiv a license to display the preprint in perpetuity. It is made available under a CC-BY 4.0 International license.

A.6.3 Requirement for transient multi-tasking

This linear analysis can be used to estimate the time interval associated with transient solutions to multi-tasking as in Fig. 3. Suppose an initial condition is set as $\boldsymbol{\kappa}(0) = c_A \boldsymbol{v}_A + c_B \boldsymbol{v}_B$. The initial condition has a component along the vector $\boldsymbol{v}_A$, which lies within the task A subspace, and a small component along $\boldsymbol{v}_B$ (that is, $|c_B| \ll |c_A|$), the eigenvector in the task B subspace associated with the largest real eigenvalue component as defined above. Suppose that $g(\|\boldsymbol{\kappa}_A(0)\|)\alpha(\boldsymbol{\sigma}_B) > 1$.

At small T, the magnitude of the projection of $\boldsymbol{\kappa}(T)$ onto the task B subspace is then approximated by $|c_B| \exp((g(\|\boldsymbol{\kappa}_A\|)\alpha(\boldsymbol{\sigma}_B) - 1)T)$. In this case, for the ratio of the projection onto the task B and task A subspaces to remain below r for a time interval of length T, the initial condition must satisfy

$$\left|\frac{c_A}{c_B}\right| > \frac{1}{r} \exp((g(\|\boldsymbol{\kappa}_A\|)\alpha(\boldsymbol{\sigma}_B) - 1)T). \tag{20}$$

Thus, increasing T requires fine-tuning of the initial condition to minimize the component along amplifying directions for task B. The degree of fine-tuning depends exponentially on $g(\|\boldsymbol{\kappa}_A\|)\alpha(\boldsymbol{\sigma}_B) - 1$. This analysis is formally valid in the limit of small r and $|c_B|$.

A.6.4 Requirement for multi-tasking through effective connectivity modulation

Eq. (19) can also be used to determine the required adjustment of the connectivity strength a_A (main-text Eq. 3) to assure transverse stability of the task A manifold when $\alpha(\boldsymbol{\sigma}_B) > \alpha(\boldsymbol{\sigma}_A)$:

$$\frac{a_A}{a_B} > \frac{\alpha(\boldsymbol{\sigma}_B)}{\alpha(\boldsymbol{\sigma}_A)}. \tag{21}$$

More generally, for networks that perform more than two tasks, the largest required adjustment equals $(\max_\mu \alpha(\boldsymbol{\sigma}_\mu))/(\min_\mu \alpha(\boldsymbol{\sigma}_\mu))$.

A.6.5 Condition for sustained activity in networks with Gaussian connectivity

For a network with Gaussian-distributed connectivity vectors, $\boldsymbol{\kappa}$ follows[16,21]:

$$\dot{\boldsymbol{\kappa}} = -\boldsymbol{\kappa} + g(\|\boldsymbol{\kappa}\|)\boldsymbol{\sigma}\boldsymbol{\kappa} = (-\boldsymbol{I} + g(\|\boldsymbol{\kappa}\|)\boldsymbol{\sigma})\,\boldsymbol{\kappa}. \tag{22}$$

We assume $g \geq 0$. We will show that if $g_{\max}\,\alpha(\boldsymbol{\sigma}) < 1$, where $g_{\max} = \max_{\boldsymbol{\kappa}} g(\|\boldsymbol{\kappa}\|)$ and $\alpha(\boldsymbol{\sigma})$ is the largest real part of the eigenvalues of $\boldsymbol{\sigma}$, then $\boldsymbol{\kappa} = \boldsymbol{0}$ is globally attracting.

Because the nonlinearity enters only as a scalar multiplying $\boldsymbol{\sigma}$, the time-dependent matrix $(-\boldsymbol{I} + g(\|\boldsymbol{\kappa}(t)\|)\boldsymbol{\sigma})$ multiplying $\boldsymbol{\kappa}(t)$ in Eq. (22) commutes for any two timepoints, and the solution can be written

$$\boldsymbol{\kappa}(t) = e^{-t}\, e^{G(t)\boldsymbol{\sigma}}\,\boldsymbol{\kappa}(0), \qquad G(t) = \int_0^t ds\; g(\|\boldsymbol{\kappa}(s)\|). \tag{23}$$

Note that $0 \leq G(t) \leq g_{\max}\, t$. This implies that $\|e^{G(t)\boldsymbol{\sigma}}\|$ grows no faster than $e^{\beta t}$ for large t, for any $\beta \in (g_{\max}\,\alpha(\boldsymbol{\sigma}), 1)$. Thus, for some constant C,

$$\lim_{t\to\infty} \|\boldsymbol{\kappa}(t)\| = \lim_{t\to\infty} \|e^{-t}e^{G(t)\boldsymbol{\sigma}}\boldsymbol{\kappa}(0)\| \leq \lim_{t\to\infty} Ce^{-(1-\beta)t}\|\boldsymbol{\kappa}(0)\| = \boldsymbol{0}. \tag{24}$$

bioRxiv preprint doi: https://doi.org/10.64898/2026.10.01.755960; this version posted October 8, 2026. The copyright holder for this preprint (which was not certified by peer review) is the author/funder, who has granted bioRxiv a license to display the preprint in perpetuity. It is made available under a CC-BY 4.0 International license.

A.6.6 Conjugate dynamics across thresholds

For a network with Gaussian-distributed connectivity vectors and a rectified-linear activation function with threshold θ_μ, that is, $\phi(x) = \max(x - \theta_\mu, 0)$, $\boldsymbol{\kappa}_\mu$ follows:

$$\dot{\boldsymbol{\kappa}}_\mu = -\boldsymbol{\kappa}_\mu + g(\|\boldsymbol{\kappa}_\mu\|, \theta_\mu)\boldsymbol{\sigma}_\mu\boldsymbol{\kappa}_\mu = -\boldsymbol{\kappa}_\mu + \frac{1}{2}\operatorname{erfc}\left(\frac{\theta_\mu}{\|\boldsymbol{\kappa}_\mu\|\sqrt{2}}\right)\boldsymbol{\sigma}_\mu\boldsymbol{\kappa}_\mu. \tag{25}$$

The second equality is derived from $g(\|\boldsymbol{\kappa}_\mu\|, \theta_\mu) = \int \mathcal{D}u\phi'(u\|\boldsymbol{\kappa}\|) = \frac{1}{2}\operatorname{erfc}\frac{\theta_\mu}{\|\boldsymbol{\kappa}_\mu\|\sqrt{2}}$.

If $\boldsymbol{\sigma}_\mu = \boldsymbol{\sigma}_\nu$, the dynamics of $\boldsymbol{\kappa}_\mu$ are conjugate to $\boldsymbol{\kappa}_\nu$ as long as θ_μ and θ_ν have the same sign, which is verified by the homeomorphism $\boldsymbol{\kappa}_\nu = \theta_\nu\boldsymbol{\kappa}_\mu/\theta_\mu$ that satisfies the conjugacy condition. This simple equivalence arises because of the threshold appearing only as a scale parameter in Eq. (25), so that flow fields associated with $\boldsymbol{\kappa}_\mu$ and $\boldsymbol{\kappa}_\nu$ are identical up to scaling by θ_ν/θ_μ. Thus, somewhat surprisingly, the threshold does not affect the qualitative dynamics of Gaussian networks with fixed $\boldsymbol{\sigma}$ unless a sign change occurs.

A.7 Effects of non-Gaussian connectivity on multi-tasking

In this section, we show that non-Gaussianity in the distributions of connectivity vector elements can change the qualitative behavior of multi-tasking solutions. To simplify the derivation, we assume each task is associated with one-dimensional bistable dynamics, like those of Task B in Fig. 3, and that $g_I = 0$. In this case, the synaptic weight matrix is:

$$J_{ij} = \frac{1}{N}\sum_{\mu\in\{A,B\}}\sigma_\mu m_i^\mu m_j^\mu, \tag{26}$$

where m_i^μ is a random i.i.d. draw from the probability distribution $P(m^\mu)$. We assume the probability distribution is the same for all tasks so that $P(m^\mu) = P(m)$ for all μ, symmetric about the origin, and normalized to have unit variance (Fig. S3A). The dynamics of this network's task variables are then:

$$\dot{\kappa}_A = -\kappa_A + \sigma_A\left\langle m^A\phi(m^A\kappa_A + m^B\kappa_B)\right\rangle, \tag{27}$$
$$\dot{\kappa}_B = -\kappa_B + \sigma_B\left\langle m^B\phi(m^A\kappa_A + m^B\kappa_B)\right\rangle. \tag{28}$$

The activation function is threshold-linear, $\phi(x) = \max(x - \theta, 0)$, with $\theta < 0$ so that activity is superthreshold. The network has a trivial fixed point at which $\kappa_A = \kappa_B = 0$. Amplification with $\sigma_\mu > 1$ is required to generate nontrivial dynamics, leading to fixed points where $\kappa_\mu^* \neq 0$, $\kappa_\nu^* = 0$ for $\mu \neq \nu$. We further require $\sigma_\mu < 2$, which ensures bounded dynamics. We focus on a nontrivial fixed point along the κ_A subspace, which is obtained by setting $\dot{\kappa}_A = 0$:

$$\kappa_A^* = \sigma_A\left\langle m\phi(m\kappa_A^*)\right\rangle. \tag{29}$$

We wish to assess how the transverse stability of this fixed point depends on the distribution $P(m)$ and on the ratio of σ_A and σ_B. The stability is determined by the eigenvalue

$$\lambda = \left.\frac{\partial\dot{\kappa}_B}{\partial\kappa_B}\right|_{(\kappa_A^*,0)} = -1 + \sigma_B\left\langle (m^B)^2\phi'(m^A\kappa_A^*)\right\rangle = -1 + \sigma_B\left\langle \phi'(m^A\kappa_A^*)\right\rangle, \tag{30}$$

where the second equality comes from the independence of m^B and m^A and the unit variance assumption.

bioRxiv preprint doi: https://doi.org/10.64898/2026.10.01.755960; this version posted October 8, 2026. The copyright holder for this preprint (which was not certified by peer review) is the author/funder, who has granted bioRxiv a license to display the preprint in perpetuity. It is made available under a CC-BY 4.0 International license.

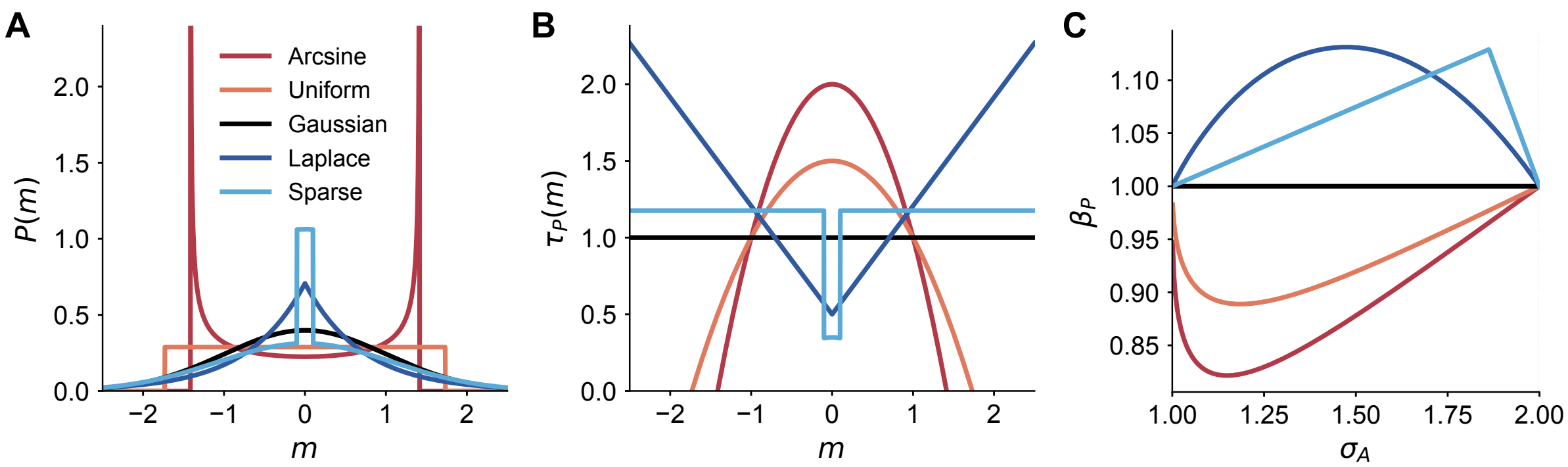

Figure S3: Effect of connectivity pattern distribution on stability.
A Probability density function $p(m)$ for five different symmetric distributions with unit variance. The arcsine distribution arises when the connectivity pattern is given by the cosine of a uniformly distributed angle: $m_i = \sqrt{2}\cos\theta_i$ for random uniform θ_i. The "sparse" distribution is a mixture of a uniform distribution $|m_i| < 0.1$ with weight 0.15, and a Gaussian distribution normalized such that the total variance is one.
B Stein kernel for the different distributions.
C Factor β_P for the different distributions as σ_A is increased. Threshold $\theta = -1$.

To express Eq. (30) in terms of the ratio of σ_A and σ_B, we derive an expression for σ_A based on the fixed point equation (Eq. 29) using Stein's identity:

$$\kappa_A^* = \sigma_A \langle m\phi(m\kappa_A^*)\rangle = \sigma_A\kappa_A^* \left\langle \tau_P(m)\, \phi'(m\kappa_A^*)\right\rangle \Rightarrow \sigma_A \left\langle \tau_P(m)\, \phi'(m\kappa_A^*)\right\rangle = 1, \tag{31}$$

where we have defined the Stein kernel of the distribution P, $\tau_P(m) = \frac{1}{p(m)}\int_m^\infty xp(x)dx$ for $p(m) > 0$ (Fig. S3B). We then define the factor

$$\beta_P = \frac{\langle \phi'(m\kappa_A^*)\rangle}{\left\langle \tau_P(m)\, \phi'(m\kappa_A^*)\right\rangle}, \tag{32}$$

which is determined by the distribution $P(m)$ and σ_A. The transverse eigenvalue can then be expressed in terms of this factor:

$$\lambda = -1 + \frac{\sigma_B}{\sigma_A}\beta_P. \tag{33}$$

The Stein kernel for a Gaussian distribution is constant, $\tau_{\mathcal{N}}(m) = 1$, implying $\beta_P = 1$. This is commonly known as Stein's lemma and often used in analyses of low-rank recurrent neural networks with Gaussian weights[16,21,30]. The argument of Sec. A.6.2, that the task subspace corresponding to the smaller value of σ_μ is transversely unstable, is an example of this.

Non-Gaussian distributions that are unimodal and concentrated around 0, such as the Laplace distribution, have Stein kernels $\tau_P(m)$ that increase with $|m|$, implying that $\beta_P > 1$ when $1 < \sigma_A < 2$ (Fig. S3C). In contrast, distributions less concentrated around 0, such as the uniform distribution or the arcsine distribution that arises from cosine-distributed weights, have Stein kernels that decrease with $|m|$, implying $\beta_P < 1$ when $1 < \sigma_A < 2$.

Suppose $\sigma_A < \sigma_B$. If $P(m)$ were Gaussian, the task A subspace would be transversely unstable. However, for distributions with $\beta_P < 1$, λ may be negative so that task A is stable even in this case. The required deviation from Gaussianity is determined by the ratio $\frac{\sigma_B}{\sigma_A}$, since the stability condition requires $\beta_P < \frac{\sigma_A}{\sigma_B} < 1$. Thus, distributions that are more dispersed than the Gaussian, such as uniform or arcsine distributions, can exhibit multistability and support sequential multi-tasking (e.g. Fig. 4).

bioRxiv preprint doi: https://doi.org/10.64898/2026.10.01.755960; this version posted October 8, 2026. The copyright holder for this preprint (which was not certified by peer review) is the author/funder, who has granted bioRxiv a license to display the preprint in perpetuity. It is made available under a CC-BY 4.0 International license.

On the other hand suppose $\sigma_A > \sigma_B$, which would imply transverse stability of the task A subspace if $P(m)$ were Gaussian. Non-Gaussian distributions that concentrate around the origin, such that $\beta_P > \frac{\sigma_A}{\sigma_B} > 1$, can destabilize both the task A and task B subspaces in this case. We found that when this occurs, the bounded dynamics converge to an attracting simultaneous multi-tasking manifold with nonzero projections onto both κ_A and κ_B.

In total, these results show that non-Gaussianity of $P(m)$ can either increase or decrease transverse eigenvalues, depending on the distribution's degree of dispersion. While the derivation was performed for one-dimensional task subspaces, we expect the qualitative results to hold for higher dimensions. The specific result that connectivity patterns whose distribution concentrates around zero generate simultaneous multi-tasking solutions motivated us to introduce connectivity barcodes, defined in the next section, as a tractable way to build multi-tasking networks.

A.8 Connectivity barcodes

In this section, we develop a mean-field theory for networks with connectivity barcodes that describes when rule inputs can select tasks sequentially or simultaneously. The previous section showed that when the distributions of the connectivity vector elements are sparse, single-task solutions tend to be destabilized. As described in the main text, one way to instantiate this mechanism is to attach a sparse barcode to each connectivity component, which means that the connectivity vectors are no longer jointly Gaussian. Let $b_i^\mu \in \{0,1\}$ be independent Bernoulli variables with mean f, and define

$$J_{ij}^\mu = \frac{b_i^\mu b_j^\mu}{Nf} \sum_{r=1}^{R_\mu} m_{\text{ind},i}^{\mu,r} n_{\text{ind},j}^{\mu,r}, \tag{34}$$

where $\{\boldsymbol{m}_{\text{ind}}^\mu, \boldsymbol{n}_{\text{ind}}^\mu\}$ are jointly Gaussian as in Sec. A.6.2. The factor $1/f$ ensures that the single-task dynamics are invariant across choices of f. The low-dimensional variables of each task are defined by the projection

$$\kappa_{r,\mu} = \frac{1}{fN} \sum_{i=1}^{N} m_i^{r,\mu} x_i = \frac{1}{fN} \sum_{i=1}^{N} b_i^\mu m_{\text{ind},i}^{r,\mu} x_i. \tag{35}$$

The single-task dynamics that would be produced if $J_{ij} = J_{ij}^\mu$ are given by Eq. (16):

$$\dot{\boldsymbol{\kappa}}_\mu = -\boldsymbol{\kappa}_\mu + g(\|\boldsymbol{\kappa}_\mu\|)\boldsymbol{\sigma}_\mu \boldsymbol{\kappa}_\mu \equiv F_\mu(\boldsymbol{\kappa}_\mu). \tag{36}$$

The mean-field dynamics for two tasks now each include a linear combination of two nonlinear terms:

$$\dot{\boldsymbol{\kappa}}_A = G_A(\boldsymbol{\kappa}_A, \boldsymbol{\kappa}_B) = -\boldsymbol{\kappa}_A + \left[(1-f)g(\|\boldsymbol{\kappa}_A\|) + fg(\|\boldsymbol{\kappa}\|)\right] \boldsymbol{\sigma}_A \boldsymbol{\kappa}_A, \tag{37}$$

$$\dot{\boldsymbol{\kappa}}_B = G_B(\boldsymbol{\kappa}_A, \boldsymbol{\kappa}_B) = -\boldsymbol{\kappa}_B + \left[(1-f)g(\|\boldsymbol{\kappa}_B\|) + fg(\|\boldsymbol{\kappa}\|)\right] \boldsymbol{\sigma}_B \boldsymbol{\kappa}_B. \tag{38}$$

The nonlinear function g is defined by the Gaussian integral $g(\|\boldsymbol{y}\|) = \int \mathcal{D}u \phi'(u\|\boldsymbol{y}\|)$, where $\|\cdot\|$ denotes the Euclidean norm, and corresponds to the average gain of neurons within the population representing the variables $\boldsymbol{y}$. The first term in each bracket comes from neurons for which $b_i^\mu = 1$ for only one task, and the second comes from neurons for which $b_i^\mu = 1$ for both tasks.

First, observe that $G_A(\boldsymbol{\kappa}_A, \mathbf{0}) = F_A(\boldsymbol{\kappa}_A)$ and $G_B(\mathbf{0}, \boldsymbol{\kappa}_B) = F_B(\boldsymbol{\kappa}_B)$, meaning that dynamics within each task subspace are conjugate to the desired task variable dynamics. Thus, the topological conjugacy requirement for multi-tasking is satisfied.

bioRxiv preprint doi: https://doi.org/10.64898/2026.10.01.755960; this version posted October 8, 2026. The copyright holder for this preprint (which was not certified by peer review) is the author/funder, who has granted bioRxiv a license to display the preprint in perpetuity. It is made available under a CC-BY 4.0 International license.

As $f \to 0$, Eqs. (37) and (38) decouple, and thus the network simultaneously multi-tasks. For the examples we consider, this transition occurs when f is small enough that all single-task subspaces lose transverse stability and lead to simultaneous multi-tasking. We first calculate this transition point, and then show how rule inputs can select individual tasks to enable sequential multi-tasking.

A.8.1 Barcodes produce single-task transverse instability and enable simultaneous multi-tasking

We will show that single-task subspaces are transversely unstable for all tasks when f is small enough. For two tasks A and B, the transverse Jacobian associated with task B's dynamics on the task A subspace is

$$\boldsymbol{T}_{AB}(\boldsymbol{\kappa}_A) = \nabla_{\boldsymbol{\kappa}_B} G_B(\boldsymbol{\kappa}_A, \mathbf{0}) = \nabla_{\boldsymbol{\kappa}_B} \left[-\boldsymbol{\kappa}_B + \left[(1-f)g(\|\boldsymbol{\kappa}_B\|) + f g(\|\boldsymbol{\kappa}\|)\right] \boldsymbol{\sigma}_B \boldsymbol{\kappa}_B\right]_{(\boldsymbol{\kappa}_A, \mathbf{0})} \tag{39}$$

$$= -\boldsymbol{I} + \left[(1-f)g(0) + f g(\|\boldsymbol{\kappa}_A\|)\right] \boldsymbol{\sigma}_B. \tag{40}$$

The maximum real part of the Jacobian's eigenvalues is therefore

$$\alpha\left(\boldsymbol{T}_{AB}(\boldsymbol{\kappa}_A)\right) = -1 + \left[(1-f)g(0) + f g(\|\boldsymbol{\kappa}_A\|)\right] \alpha(\boldsymbol{\sigma}_B). \tag{41}$$

We assume that $g(0)\alpha(\boldsymbol{\sigma}_B) > 1$, which ensures absence of a stable trivial fixed point at the origin. Then, for any point $\boldsymbol{\kappa} = (\boldsymbol{\kappa}_A, \mathbf{0})$ on the task A subspace for which $g(\|\boldsymbol{\kappa}_A\|) < g(0)$, there is a value of f below which this point is transversely unstable. The assumption $g(\|\boldsymbol{\kappa}_A\|) < g(0)$ is true for networks in which the activation function threshold $\theta < 0$, which is necessary for sustained activity. This value of f is given by

$$f < \frac{g(0) - (\alpha(\boldsymbol{\sigma}_B))^{-1}}{g(0) - g(\|\boldsymbol{\kappa}_A\|)}. \tag{42}$$

We now consider the specific case in which each task subspace has an attractor $\boldsymbol{\kappa}^*_\mu(t)$ on which $\|\boldsymbol{\kappa}^*_\mu\|$ is constant (for example, a fixed point or a circular limit cycle), and we evaluate transverse stability on this attractor. In this case, $g(\|\boldsymbol{\kappa}^*_\mu\|) = 1/\alpha(\boldsymbol{\sigma}_\mu)$. This yields the criterion

$$f < \frac{\alpha(\boldsymbol{\sigma}_A)(g(0)\alpha(\boldsymbol{\sigma}_B) - 1)}{\alpha(\boldsymbol{\sigma}_B)(g(0)\alpha(\boldsymbol{\sigma}_A) - 1)}. \tag{43}$$

An identical argument holds for the task B subspace. In simulations, we have found that when all single task solutions are transversely unstable and the dynamics do not grow unboundedly (e.g., with a bounded nonlinearity, or $\alpha(\boldsymbol{\sigma}) < 2$ for threshold-linear neurons) there is a manifold that supports the simultaneous performance of tasks A and B. For networks with more than two tasks, if Eq. 42 holds for all pairs of tasks, all task subspaces are transversely unstable.

A.8.2 Rule inputs in barcoded networks enable sequential multi-tasking

We have shown that barcodes produce simultaneous multi-tasking in networks without external input. We now show that it is possible to construct networks with barcodes in which rule inputs select specific tasks, enabling either sequential or simultaneous multi-tasking. We assume that the activation function is rectified-linear, $\phi(x) = \max(x - \theta, 0)$, but, unlike the previous section, we assume that $\theta > 0$, so that in the absence of an input, the network is silent. We then apply a rule input proportional to the barcode of task A:

$$\dot{x}_i = -x_i + \sum_{\mu \in \{A,B\}} \sum_{j=1}^{N} J^{\mu}_{ij} \phi(x_j) + I^A_i, \tag{44}$$

$$I^A_i = s b^A_i, \tag{45}$$

bioRxiv preprint doi: https://doi.org/10.64898/2026.10.01.755960; this version posted October 8, 2026. The copyright holder for this preprint (which was not certified by peer review) is the author/funder, who has granted bioRxiv a license to display the preprint in perpetuity. It is made available under a CC-BY 4.0 International license.

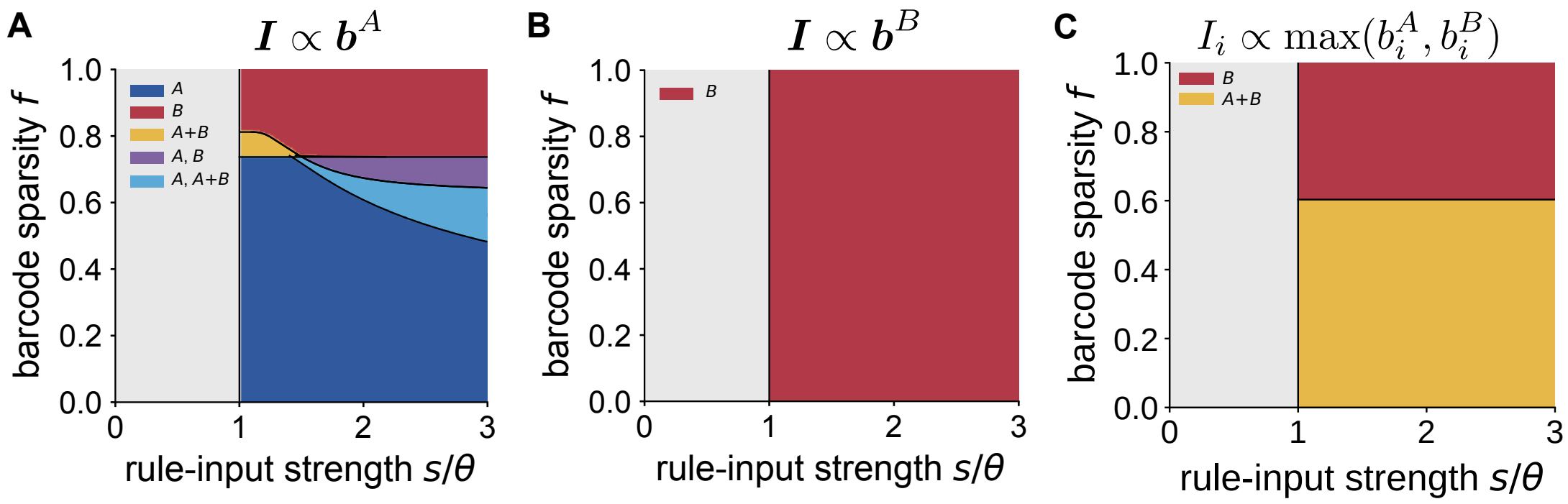

Figure S4: Barcoded rule input in a network with $\alpha(\boldsymbol{\sigma}_B) > \alpha(\boldsymbol{\sigma}_A)$.
A Phase diagram showing stable task manifolds as a function of the barcode sparsity and the strength of the rule input. The input is proportional to the task A barcode. If Eq. 51 is satisfied, task A is selected (blue region). Strong inputs may lead to additional stable manifolds. A+B denotes simultaneous multi-tasking. A, B denotes multistability of task A and B solutions. A, A+B denotes multistability of a task A solution and a simultaneous multi-tasking solution.
B Similar to panel A, when the input is proportional to the task B barcode.
C Similar to panels A and B, when the input is given by both task's barcodes.
Task parameters: $\alpha(\boldsymbol{\sigma}_A) = 1.4$ and $\alpha(\boldsymbol{\sigma}_B) = 1.9$.

Activity $\boldsymbol{x}(t)$ can be parameterized with task variables $\boldsymbol{\kappa}_\mu(t)$, which represent projections along the vectors $\{\boldsymbol{m}^{\mu,r}\}$ as in Eq. 3, together with an additional variable κ_I to parameterize activity projected onto the vector $\boldsymbol{b}^A$. The dynamics of these latent variables are

$$\dot{\kappa}_I = -\kappa_I + s, \tag{46}$$
$$\dot{\boldsymbol{\kappa}}_A = -\boldsymbol{\kappa}_A + [(1-f)g(\kappa_I, \|\boldsymbol{\kappa}_A\|) + fg(\kappa_I, \|\boldsymbol{\kappa}\|)]\, \boldsymbol{\sigma}_A \boldsymbol{\kappa}_A \equiv G_A(\boldsymbol{\kappa}_A, \boldsymbol{\kappa}_B, \kappa_I), \tag{47}$$
$$\dot{\boldsymbol{\kappa}}_B = -\boldsymbol{\kappa}_B + [(1-f)g(0, \|\boldsymbol{\kappa}_B\|) + fg(\kappa_I, \|\boldsymbol{\kappa}\|)]\, \boldsymbol{\sigma}_B \boldsymbol{\kappa}_B \equiv G_B(\boldsymbol{\kappa}_A, \boldsymbol{\kappa}_B, \kappa_I). \tag{48}$$

The nonlinear function g is defined by the Gaussian integral $g(\mu, \|\boldsymbol{y}\|) = \int \mathcal{D}u \phi' (\mu + u\|\boldsymbol{y}\|)$, where μ describes the average current to each neural population.

Activation of a selected task. We first show that, as long as the input strength s is larger than the single neuron threshold, $s > \theta$, and the barcode is sparse enough, the multi-tasking network generates an attracting single-task subspace. We assume that the dynamics F_μ associated with the performance of task μ are again given by Eq. (36), corresponding to the dynamics of a single-task network. The activation function for neurons in this single-task network has a threshold that we denote by $\theta_1 < 0$.

In the barcoded network, the dynamics on the task A subspace are determined by Eq. (47) when $\boldsymbol{\kappa} = (\boldsymbol{\kappa}_A, \mathbf{0})$. In this subspace, the dynamics do not depend on the value of f and have the same form as the desired single-task dynamics F_A, except that the effective activation function threshold is different and given by $\theta - s$. The result of Sec. A.6.6 implies that as long as $s > \theta$, the barcoded network's dynamics are conjugate to those defined by F_A. Thus, a relatively unrestrictive condition on the magnitude of the rule input is sufficient to produce the desired dynamics.

Next, to assess stability of the task A subspace, we evaluate the largest real part of the transverse eigenvalues:

$$\alpha\left(\boldsymbol{T}_{AB}(\boldsymbol{\kappa}_A)\right) = -1 + [(1-f)g(0,0) + fg(s, \|\boldsymbol{\kappa}_A\|)]\, \alpha(\boldsymbol{\sigma}_B) \tag{49}$$
$$= -1 + fg(s, \|\boldsymbol{\kappa}_A\|)\, \alpha(\boldsymbol{\sigma}_B), \tag{50}$$

bioRxiv preprint doi: https://doi.org/10.64898/2026.10.01.755960; this version posted October 8, 2026. The copyright holder for this preprint (which was not certified by peer review) is the author/funder, who has granted bioRxiv a license to display the preprint in perpetuity. It is made available under a CC-BY 4.0 International license.

where the second line comes from the assumption of a positive threshold of the activation function, implying $g(0,0) = 0$. Because $g \leq 1$, transverse stability is therefore always guaranteed if $f < 1/\alpha(\boldsymbol{\sigma}_B)$. More generally, in networks that perform more than two tasks, if $f < 1/\alpha(\boldsymbol{\sigma}_\mu)$ for all μ, all task subspaces are transversely stable. Thus, we have established topological conjugacy and attractiveness of task subspaces when a rule input is provided, if f is small enough.

We now derive a less restrictive bound on f for specific single-task dynamics, such as fixed points or limit cycles, that are generated by an instability when $\boldsymbol{\kappa}_\mu$ approaches zero. For such tasks, the dynamics stabilize when the amplifying dynamics are balanced by the average neuronal gain: $g(\|\boldsymbol{\kappa}_\mu\|)\alpha(\boldsymbol{\sigma}_\mu) = 1$. Substituting this into Eq. (50) leads to the following condition on f:

$$f < \frac{\alpha(\boldsymbol{\sigma}_A)}{\alpha(\boldsymbol{\sigma}_B)}. \tag{51}$$

An example phase diagram for a network with $\alpha(\boldsymbol{\sigma}_A) < \alpha(\boldsymbol{\sigma}_B)$ and receiving a rule input proportional to the task A barcode is shown in Fig. S4A. The condition of Eq. (51) is satisfied in the blue, cyan, and purple regions. In these regions an attracting task A subspace exists (although in the purple and cyan regions, another multistable solution also exists, see next section). On the other hand, the same network receiving a task B barcode rule input only performs task B, since $\alpha(\boldsymbol{\sigma}_A) < \alpha(\boldsymbol{\sigma}_B)$ (Fig. S4B).

Complete phase diagram. We next analyze under what situations a network receiving a rule input proportional to the task A barcode also generates dynamics in the task B subspace (Fig. S4A, red, yellow, purple, and cyan regions). These solutions can be viewed as spurious states in which an input intended to select task A can select another task or combination of tasks. As in the derivation of Eq. (51), we assume that each task is associated with an attractor $\boldsymbol{\kappa}^*_\mu(t)$ on which $g(\|\boldsymbol{\kappa}^*_\mu\|)\alpha(\boldsymbol{\sigma}_\mu) = 1$. Such solutions can be characterized analytically in the limit $s = \theta + \epsilon$, $\epsilon \to 0$.

Based on Eq. (48), when s is above threshold but small enough such that $g(0, \|\boldsymbol{\kappa}^*_B\|) \to 0$, activity in the task B subspace is nondecaying only if $f > 1/\alpha(\boldsymbol{\sigma}_B)$. Transverse stability of the task B subspace is determined by:

$$\alpha\left(\nabla_{\boldsymbol{\kappa}_A} G_A(\mathbf{0}, \boldsymbol{\kappa}^*_B, s)\right) = -1 + \left[(1-f)g(s,0) + fg(s, \|\boldsymbol{\kappa}^*_B\|)\right]\alpha(\boldsymbol{\sigma}_A). \tag{52}$$

The gain factor $g(s,0) = 1$ for $s > \theta$. Additionally, the task dynamics impose $fg(s, \|\boldsymbol{\kappa}^*_B\|) = 1/\alpha(\boldsymbol{\sigma}_B)$, so that task B is stable only if

$$f > 1 - \frac{\alpha(\boldsymbol{\sigma}_B) - \alpha(\boldsymbol{\sigma}_A)}{\alpha(\boldsymbol{\sigma}_B)\alpha(\boldsymbol{\sigma}_A)}. \tag{53}$$

Note that this can only be satisfied if $\alpha(\boldsymbol{\sigma}_B) > \alpha(\boldsymbol{\sigma}_A)$. This shows that, if task B is more amplifying than task A and f is too large, a rule input intended to select task A can instead select task B (Fig. S4A, red region).

Next, we examine whether a simultaneous multi-tasking solution exists, i.e. $\|\boldsymbol{\kappa}^*_A\| \neq 0$ and $\|\boldsymbol{\kappa}^*_B\| \neq 0$. Using Eq. (48), such a solution requires $fg(s, \|\boldsymbol{\kappa}^*\|) = \alpha(\boldsymbol{\sigma}_B)^{-1}$. Introducing this result into Eq. (47), we obtain the condition $(1-f)g(s, \|\boldsymbol{\kappa}_A\|) = \alpha(\boldsymbol{\sigma}_A)^{-1} - \alpha(\boldsymbol{\sigma}_B)^{-1}$. Such solutions require $g(s, \|\boldsymbol{\kappa}^*\|) < g(s, \|\boldsymbol{\kappa}^*_A\|) < 1$. Combining these three expressions, we obtain a range of sparsity values for which the simultaneous multi-tasking solution exists (Fig. S4A, yellow region):

$$\frac{\alpha(\boldsymbol{\sigma}_A)}{\alpha(\boldsymbol{\sigma}_B)} < f < 1 - \frac{1}{\alpha(\boldsymbol{\sigma}_A)} + \frac{1}{\alpha(\boldsymbol{\sigma}_B)}. \tag{54}$$

bioRxiv preprint doi: https://doi.org/10.64898/2026.10.01.755960; this version posted October 8, 2026. The copyright holder for this preprint (which was not certified by peer review) is the author/funder, who has granted bioRxiv a license to display the preprint in perpetuity. It is made available under a CC-BY 4.0 International license.

This range coincides with the range in which both the task A and B subspaces are unstable (Eqs. 51 and 53). Evaluation of the Jacobian associated with this simultaneous multi-tasking solution shows that, if the solution exists, it is always stable.

The full phase diagram, for arbitrary s, can be assessed numerically. As the input strength increases further from $s \gtrsim \theta$, multistable phases also appear, where both the task A and task B attractors are stable, or even multistable phases where both a single task A attractor and a simultaneous task A and B attractor are stable (Fig. S4A, purple and cyan regions). These phase boundaries are calculated by numerically finding roots to the existence and transverse stability equations.

Combination of rule inputs for simultaneous multi-tasking. We have shown it is possible to select task A (Fig. S4A, blue region) or task B (Fig. S4B, red region) with a rule input proportional to the task barcodes. To select tasks μ and ν simultaneously, we provide a rule input that combines two barcodes, $I_i = s\max(b_i^\mu, b_i^\nu)$. If the network performs only two tasks A and B, the input is received by all neurons with $b_i^A \neq 0$ or $b_i^B \neq 0$ homogeneously, corresponding to an effective change in these neurons' mean input. Therefore, the network simultaneously multi-tasks if f is below the critical value given by Eq. (42) (Fig. S4C).

A.8.3 Barcodes with non-binary elements

We now consider a barcode model with

$$m_i^{\mu,r} = (\delta + (1-\delta)b_i^\mu) \cdot m_{\text{ind},i}^{\mu,r}, \qquad n_i^{\mu,r} = b_i^\mu \cdot n_{\text{ind},i}^{\mu,r}. \tag{55}$$

If $\delta > 0$, neurons that do not drive the dynamics of a task (that is, neurons i for which $b_i^\mu = 0$ and thus $n_i^{\mu,r} = 0$) are nonetheless tuned to that task. The magnitude of δ controls the strength of this tuning, with $\delta = 0$ reducing to the binary barcode model of the previous section and $\delta = 1$ corresponding to equal tuning to all tasks. The low-dimensional variables are rescaled to account for this parameter so that the single-task dynamics remain unchanged:

$$\kappa_{r,\mu} = \frac{1}{N(f + (1-f)\delta^2)} \sum_{i=1}^{N} b_i^\mu m_{\text{ind},i}^{r,\mu} x_i. \tag{56}$$

The dynamics in a two-task network, analogous to Eqs. (37) and (38), are:

$$\dot{\boldsymbol{\kappa}}_A = -\boldsymbol{\kappa}_A + \left[(1-f)g(\sqrt{\|\boldsymbol{\kappa}_A\|^2 + \delta^2\|\boldsymbol{\kappa}_B\|^2}) + fg(\|\boldsymbol{\kappa}\|)\right] \boldsymbol{\sigma}_A \boldsymbol{\kappa}_A, \tag{57}$$

$$\dot{\boldsymbol{\kappa}}_B = -\boldsymbol{\kappa}_B + \left[(1-f)g(\sqrt{\|\boldsymbol{\kappa}_B\|^2 + \delta^2\|\boldsymbol{\kappa}_A\|^2}) + fg(\|\boldsymbol{\kappa}\|)\right] \boldsymbol{\sigma}_B \boldsymbol{\kappa}_B. \tag{58}$$

We consider a network with $\theta < 0$, and $1 < \alpha(\boldsymbol{\sigma_B}) < \alpha(\boldsymbol{\sigma_A}) < 2$. The maximum real part of the transverse eigenvalues for the task A subspace is

$$\alpha\left(\boldsymbol{T}_{AB}(\boldsymbol{\kappa}_A)\right) = -1 + \left[(1-f)g(\delta\|\boldsymbol{\kappa}_A\|) + fg(\|\boldsymbol{\kappa}_A\|)\right]\alpha(\boldsymbol{\sigma}_B). \tag{59}$$

For tasks with constant norm, $g(\|\boldsymbol{\kappa}_A^*\|)\alpha(\boldsymbol{\sigma}_A) = 1$. Under this assumption, the sparsity criterion that ensures the single-task solution is unstable is:

$$f < \frac{\alpha(\boldsymbol{\sigma}_A)(g(\delta\|\boldsymbol{\kappa}_A^*\|)\alpha(\boldsymbol{\sigma}_B) - 1)}{\alpha(\boldsymbol{\sigma}_B)(g(\delta\|\boldsymbol{\kappa}_A^*\|)\alpha(\boldsymbol{\sigma}_A) - 1)}, \tag{60}$$

bioRxiv preprint doi: https://doi.org/10.64898/2026.10.01.755960; this version posted October 8, 2026. The copyright holder for this preprint (which was not certified by peer review) is the author/funder, who has granted bioRxiv a license to display the preprint in perpetuity. It is made available under a CC-BY 4.0 International license.

which can be solved numerically. As in previous sections, when the task A subspace is transversely unstable, a simultaneous multi-tasking solution emerges.

The criterion in Eq. (60) shows that there is a trade-off between the selectivity of neurons to tasks whose dynamics they do not drive, described by δ, and the barcode sparsity f. The maximum f at which simultaneous multi-tasking is achieved corresponds to $\delta = 0$, and is determined by Eq. (43). As δ increases, f must decrease to permit simultaneous multi-tasking. In the limit $f \to 0$, the maximum δ that permits simultaneous multi-tasking is:

$$\delta < \delta_{\max} = \frac{g^{-1}(1/\alpha(\boldsymbol{\sigma_B}))}{g^{-1}(1/\alpha(\boldsymbol{\sigma_A}))}, \tag{61}$$

where g^{-1} denotes the inverse of the average gain function.

A.9 Global inhibition in threshold-linear networks

Unbounded single neuron activation functions, such as rectified-linear, can lead to instability for strongly amplifying dynamics. In particular, if $\alpha(\boldsymbol{\sigma}_\mu) > 2$, amplification cannot be compensated by the gain term $g(\|\boldsymbol{\kappa}_\mu\|)$, which in this case has a lower bound of $1/2$ for $\theta < 0$. One solution to such instability is the addition of global inhibition.

Following the main text, we consider an inhibitory population that integrates activity across all neurons in the network and provides negative feedback with strength g_I (Eq. 2). The dynamics of the latent variables, parameterized in Eq. (3), are then:

$$\dot{\bar{x}} = -\bar{x} - g_I \langle \phi \rangle, \tag{62}$$

$$\dot{\boldsymbol{\kappa}}_A = G_A(\bar{x}, \boldsymbol{\kappa}_A, \boldsymbol{\kappa}_B) \tag{63}$$

$$\dot{\boldsymbol{\kappa}}_B = G_B(\bar{x}, \boldsymbol{\kappa}_A, \boldsymbol{\kappa}_B), \tag{64}$$

where $\langle \phi \rangle$ indicates the average firing rate.

For networks with inhibition, the trivial fixed point is defined by $\boldsymbol{\kappa}_A = \boldsymbol{\kappa}_B = \mathbf{0}$ and $\theta < \bar{x}_0 < 0$, where $\bar{x}_0$ satisfies $\bar{x}_0 = -g_I \phi(\bar{x}_0)$. At this fixed point, the variable $\bar{x}$ changes the effective threshold of the nonlinearity from θ to $\theta - \bar{x}(t)$. Nevertheless, since $\bar{x} > \theta$, the effective threshold does not change sign, and the result of Sec. A.6.6 implies that the dynamics in the vicinity of the trivial fixed point remain conjugate to the dynamics without inhibition. A similar effect is observed for task-related dynamics, as long as $\bar{x}(t) > \theta$. For the fixed-point and limit-cycle tasks we have considered, $\bar{x}(t)$ is larger than the threshold if $\alpha(\boldsymbol{\sigma}_\mu) < 2$, so that multi-tasking solutions are qualitatively unchanged by the presence of inhibition.

However, if $\alpha(\boldsymbol{\sigma}_\mu) > 2$, $\bar{x}$ becomes small enough that the effective threshold changes sign to compensate the amplifying task dynamics. In this parameter range, task dynamics are stabilized by global inhibition, and this affects solutions qualitatively. For example, for non-Gaussian connectivity vectors, the factor β_P (Eq. 32; Fig. S3) crosses the unity line when $\alpha(\boldsymbol{\sigma}_\mu) = 2$. Therefore, a non-Gaussian distribution that generates multistable task manifolds without inhibition will generate a simultaneous multi-tasking manifold in the inhibition-dominated regime. Conversely, for non-Gaussian distributions that lead to simultaneous multi-tasking, such as connectivity barcodes, the inhibition-dominated regime leads instead to multistability.

For barcoded connectivity, intuition is provided by noting that in the absence of inhibition, barcodes lead to simultaneous multi-tasking when they are sparse enough because specialized neural

bioRxiv preprint doi: https://doi.org/10.64898/2026.10.01.755960; this version posted October 8, 2026. The copyright holder for this preprint (which was not certified by peer review) is the author/funder, who has granted bioRxiv a license to display the preprint in perpetuity. It is made available under a CC-BY 4.0 International license.

populations encode different tasks with little interference. The intuition is reversed in networks stabilized by global inhibition: whenever one population is active, feedback inhibition silences all others, leading to multistability.

In conclusion, global inhibition can stabilize networks with threshold-linear activation functions and affects their multi-tasking properties. In particular, changes in inhibition can cause a network that simultaneously multi-tasked to sequentially multi-task via multistable dynamics. Alternative inhibitory mechanisms, such as task-specific inhibition, will also likely have different effects on multi-tasking.

A.10 Effects of activation function

Most theories of nonlinear networks with low-rank connectivity have used neuron models with bounded sigmoid activation functions such as $\phi(x) = \tanh(x)$, though many results do not depend on this choice. Here, we have focused on threshold-linear functions, which are consistent with the presence of a spike threshold and inter-neuronal communication via nonnegative firing rates.

The mean-field description of networks with low-rank connectivity applies to any pointwise nonlinear activation function. In this description, the dynamics of single-task networks are given by Eq. (16). The scalar nonlinear function g, which represents the average gain across the network, depends on the nonlinearity. However, for any non-decreasing activation function, including threshold-linear and sigmoid functions, Gaussian connectivity vectors lead to transverse instability.

Our results on the effects of non-Gaussian connectivity in Sec. A.7 also extend to networks with sigmoid activation functions. Indeed, whether the factor β_P (Eq. 32) is above or below one, which determines whether networks exhibit multistability or support simultaneous multi-tasking, depends on how $\phi'(m\kappa^*)$ behaves as $|m|$ increases. For both hyperbolic tangent and threshold-linear activation functions in networks with $1 < \alpha(\boldsymbol{\sigma}) < 2$, the gain decreases as $|m|$ increases. Nevertheless, as $\alpha(\boldsymbol{\sigma})$ approaches 2, for the former and unlike the latter case, the factor β_P does not approach one, but remains above or below one depending on the distribution.

The sparsity criterion that determines when networks simultaneously multi-task (Eq. 43) also holds for networks with sigmoid activation functions. An important difference between networks with different activation functions appears when using rule inputs in barcoded networks (Sec. A.8.2). For threshold-linear networks, rule inputs drive specific neuronal subpopulations above threshold, while other neurons remain below threshold. Networks with sigmoid activation functions do not have a threshold, but can implement a similar mechanism. Strong inputs, both positive and negative, decrease the gain of a population by driving its neurons towards saturation.

One implementation of rule inputs in networks with sigmoid activation functions provides a background input negatively correlated with all barcodes: $\boldsymbol{I}_0 = -s_0 \sum_\mu \boldsymbol{b}_\mu$, which broadly suppresses task-related dynamics. To select task μ, an additional rule input correlated with task μ's barcode is provided, $\boldsymbol{I}_\mu = s\boldsymbol{b}_\mu$. The complete quantitative phase diagram, and its behavior as s varies, will depend on the specific activation function.
